# Localization in semi-infinite herringbone waveguides

**DOI:** 10.1098/rspa.2017.0590

**Published:** 2018-03-28

**Authors:** S. G. Haslinger, I. S. Jones, N. V. Movchan, A. B. Movchan

**Affiliations:** 1 Department of Mathematical Sciences, University of Liverpool, Liverpool L69 7ZL, UK; 2 Mechanical Engineering and Materials Research Centre, Liverpool John Moores University, Liverpool L3 3AF, UK

**Keywords:** flexural waves, platonic crystals, localization, herringbone systems, Kirchhoff plate

## Abstract

The paper includes novel results for the scattering and localization of a time-harmonic flexural wave by a semi-infinite herringbone waveguide of rigid pins embedded within an elastic Kirchhoff plate. The analytical model takes into account the orientation and spacing of the constituent parts of the herringbone system, and incorporates dipole approximations for the case of closely spaced pins. Illustrative examples are provided, together with the predictive theoretical analysis of the localized waveforms.

## 1. Introduction

Herringbone systems are a source of great interest in the scientific community across a broad spectrum of fields encompassing branches of physics, chemistry and biology. In crystallography and solid-state physics, the preference for herringbone-type close packing of crystals has long been a topic of study for many groups of researchers (see, for example, the article by Arlt & Sasko [1] concerning the domain configuration in barium titanate ceramics).

The subject of domain structure to minimize the energy of crystals and grains is still highly relevant today in modern technologies involving ferroelectric and piezoelectric materials. A formal classification of all the rank-2 laminate arrangements for a ferroelectric single crystal was given in [2], with half featuring herringbone patterns on at least one surface. The patterns are significant in such materials because the geometric arrangement of domains influences both the macroscopic and microscopic properties, including elastic moduli and dielectric permittivity. The characteristic switching behaviour of a ferroelectric is also strongly dependent on the domain pattern [3].

Another related application area is the study of organic semiconductors (OSCs), which are used in organic field effect transistors, organic solar cells and organic LEDs [4]. All of these devices are dependent on the solid-state packing of the OSCs, with, for example, the charge transport of the organic field effect transistors being one important property governed by the molecular packing arrangement [5].

**Figure 1.**
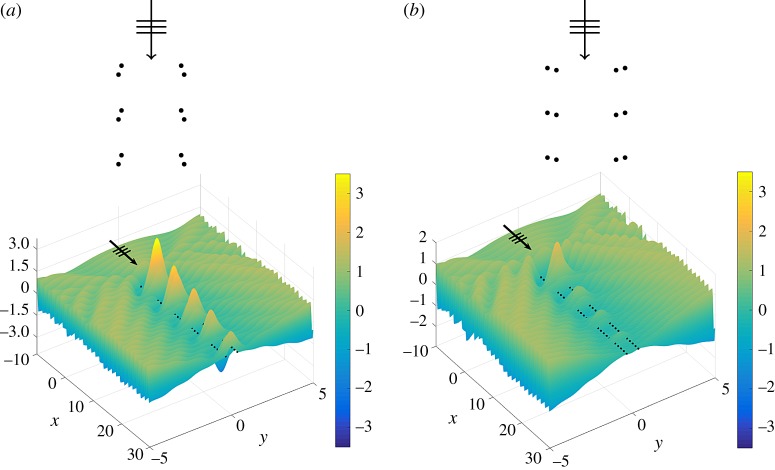
Displacement fields for two flexural herringbone waveguides with all parameters prescribed identically except for the orientation of the dipoles, for the case of normal incidence. The pairs are aligned to form (*a*) a convex entrance and (*b*) a concave entrance. For an aluminium plate of thickness 5 mm, the frequency is 23.3 Hz in both cases. (Online version in colour.)

In this paper, we design and model a novel herringbone system constructed within an elastic Kirchhoff plate. We show how the parameters governing the herringbone geometry may be tuned to optimize waveguiding and localization of flexural waves within a structured plate (figure 1). In a similar way to the herringbone arrangement of crystals being favourable for thin film transport [6], we show that the concept of cladding a simple two-grating waveguide with an additional pair of appropriately located gratings enhances the waveguiding effect. Adopting a wave scattering method and a dipole approximation assumption, we demonstrate an elegant mathematical formulation and solution to the scattering of flexural plane waves by an elastic plate pinned in a herringbone fashion.

In recent years, there has been substantial interest in wave propagation in structured elastic plates, motivated by the abundance of potential applications in engineering and materials science. The papers featuring wave scattering in the Kirchhoff model [7] and the Mindlin model [8] were important contributions to the modelling of the dynamic response of thin elastic plates containing impurities. The ability to customize systems to control the direction and amplification of flexural waves is important for the design of metamaterials and microstructured systems that possess special properties unattainable with natural materials. Recently, scattering of flexural waves in the context of metamaterial applications was discussed in [9,10].

An attraction of structured Kirchhoff plates is that many of the methods and ideas associated with photonic crystals can be applied to platonic crystals [11]. The fourth-order biharmonic operator introduces mathematical subtleties to the analysis of the scattering of flexural waves compared with the usual second-order derivatives for the wave equation of optics and acoustics, but with an added advantage. The biharmonic two-dimensional Green’s function is bounded, rather than diverging logarithmically at the source point (as in the case of the Helmholtz operator). This feature is of particular importance for the special case of periodically pinned elastic plates.

Numerous methods have been implemented, including Fourier series expansions by, among others, Mead (see [12] for a review), and the use of Green’s functions by Evans & Porter [13] and Smith *et al.* [14], and more recently by Antonakakis and co-workers [15,16]. A complementary approach using multipole methods has been employed in a series of papers [11,17–20], where the limiting case of small holes with a clamped edge was considered, recovering the case of rigid pins. In the articles [18,19], structured plates containing a finite number of infinite gratings were considered, and their ability to trap and localize flexural wave energy was analysed. The analysis of semi-infinite grating stacks in this article uses some similar ideas but demonstrates several concepts and effects unique to a semi-infinite system, as will be explained below.

Bloch–Floquet analysis for an elementary cell is a common approach to the modelling of infinite periodic systems, but for a semi-infinite platonic crystal (an infinite plate containing a semi-infinite periodic array, not to be confused with a pinned semi-infinite plate as in, for example, [21]) this technique is no longer directly applicable. Recent analysis for flexural wave scattering has been conducted by Haslinger *et al.* [22,23] and Jones *et al.* [24]. The methods of solution adopted by these authors included a discrete Wiener–Hopf method and a wave scattering technique inspired by the classical papers of, respectively, Hills & Karp [25] and Foldy [26], for related problems of two-dimensional membrane waves in discrete semi-infinite clusters. A review of the problem of acoustic scattering by a two-dimensional semi-infinite periodic array of isotropic point scatterers is provided by Linton & Martin [27].

There has also been extensive interest in the scattering of plane waves by semi-infinite crystals in electromagnetism. Early investigations include the detailed coverage provided by Mahan & Obermair [28] and Mead [29], where nearest-neighbour and dielectic approximations were analysed and discussed. More recently, research has been conducted for applications in the design of metamaterials, for example in [30], where a point dipole approximation for sufficiently small scatterers was implemented. In addition, Belov & Simovski [30] used knowledge of the eigenmodes for infinite crystals to give insight on the problems of scattering of plane waves by analogously composed semi-infinite crystals. Another recent study [31] considered the wave dynamics at the interface of a homogeneous half-space and a half-space of plasmonic nanospheres, using a discrete Wiener–Hopf technique incorporating the assumption that each nanosphere may be described by the single dipole approximation.

This paper addresses the scattering of flexural plane waves propagating in a structured Kirchhoff plate. A novel design depicts a waveguide consisting of a pair of pinned gratings, which is augmented by an extra pair of gratings, each of which is positioned exterior, but close, to the original set. Defining shift vectors for each of the upper and lower pairs, a herringbone system is constructed. A natural configuration to consider is a regular double-pinned structure (symmetric herringbone). One may also classify the special cases for which the leading pair of gratings is either the inner pair (convex entrance) or the outer pair (concave entrance), as illustrated in figure 1. The twin parameters of magnitude and orientation of the shift vectors are used to design herringbone systems to guide and direct waves.

The proximity of the constituent members of the shifted pairs promotes the use of a dipole approximation for the pairs of closely spaced pins. The first part of the paper analyses the case of a shifted pair of semi-infinite gratings in detail, with an emphasis on using the dipole approximation. This idea is taken further by considering the replacement of each of the dipole pairs by an array of points with two prescribed boundary conditions, zero displacement and zero directional derivative.

We first consider the problem of a single shifted pair in §2a,b, followed by the semi-infinite line array of sources and dipoles in §2c. Illustrative examples and comparisons of the two approaches are demonstrated in §2d. We then formulate the problem for the herringbone system, in both its exact form in §3a and with the dipole approximations in §3b,c. We illustrate the waveguiding and localization capabilities, combining the concepts of the dipole approximation and waveguide analysis in §3d. The latter method recalls the ideas used for both the infinite grating waveguide in [19] and the semi-infinite waveguide in [24]. The former paper incorporates the eigenvalue problem for finite stacks of shifted infinite gratings, while the latter article uses a similar technique to identify blocking and trapping regimes for a pair of parallel semi-infinite gratings. Concluding remarks are drawn together in §4.

## 2. Kirchhoff plate with a pair of shifted semi-infinite rows of pins

**Figure 2.**
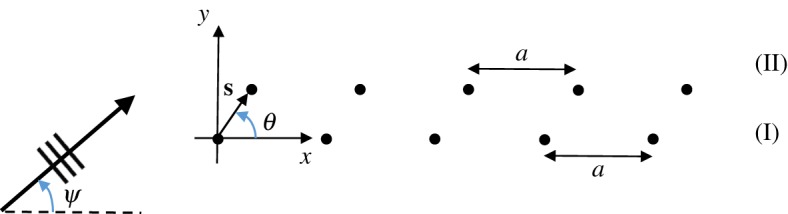
Two semi-infinite horizontal lines of rigid pins with spacing *a* in an elastic Kirchhoff plate. A plane wave is incident at an angle *ψ* and the lines are shifted relative to one another according to the shift vector **s** with orientation *θ*. (Online version in colour.)

We introduce a model problem for a pair of shifted semi-infinite rows of rigid pins embedded in a Kirchhoff elastic plate (figure 2). Two-dimensional axes are chosen as shown and one line of pins is shifted relative to the other by the shift vector **s**=*s*_1_**i**+*s*_2_**j**. The horizontal spacing between the pins in a single grating is represented by *a*>|**s**|>0. A plane wave is incident at an angle *ψ* on the lines of pins, which occupy the positive half-plane *x*≥0 (figure 2).

### (a) Algebraic system

In the time-harmonic regime, the amplitude *u* of the flexural displacement of a homogeneous Kirchhoff plate satisfies the governing equation

2.1 $$\Delta^{2}u-\beta^{4}u=0,$$

with *β*^4^=*ρhω*^2^/*D*; here *h* is the plate thickness, *ρ* is its density (mass per unit volume), *ω* is the radian frequency and *D*=*Eh*^3^/(12(1−*ν*^2^)) is the flexural rigidity of the plate; *E* and *ν* are the Young modulus and the Poisson ratio of the plate, respectively.

Consider an incident field at the point **r**=(*x*,*y*) whose amplitude *u*_inc_(**r**) is given by

2.2 $$u_{\mathrm{inc}}(\text{r})=u_{\mathrm{inc}}(x,y)=e^{i\beta (x\cos\psi +y\sin\psi )},\;|u_{\mathrm{inc}}|=1.$$

In the two-dimensional case, the single-source Green’s function satisfying the equation

2.3 $$\Delta^{2}g(\beta ;\text{r};\text{r'})-\beta^{4}g(\beta ;\text{r};\text{r'})=\delta (\text{r}-\text{r'})$$

is expressed as

2.4 $$g(\beta ;\text{r};\text{r'})=\frac{i}{8\beta^{2}}\left[H_{0}^{\left(1\right)}(\beta |\text{r}-\text{r'}|)+\frac{2i}{\pi }K_{0}(\beta |\text{r}-\text{r'}|)\right],$$

with respect to the source point **r’**=(*x*′,*y*′). The Green’s function is finite at this point, rather than diverging logarithmically, as is the case for the two-dimensional Helmholtz operator. This non-singular property is useful in the wave scattering approach we employ, whereby the scattered field is expressed as a sum of Green’s functions, a method widely reported for acoustics by, among others, [25–27].

The total field is represented by the superposition of the incident and scattered fields, taking into account all of the scatterers whose unknown intensities are to be determined. Thus, the total flexural displacement *u*(*x*,*y*) for the pair of platonic gratings is expressed as

2.5 $$u(x,y)=u_{\mathrm{inc}}(x,y)+\sum_{n=0}^{\infty }A_{n}^{\left(I\right)}g(\beta ;x,y;na,0)+\sum_{m=0}^{\infty }A_{m}^{\left(\mathrm{II}\right)}g(\beta ;x,y;s_{1}+ma,s_{2}),$$

where the scattered field coefficients $A_{n}^{\left(I\right)}$ and $A_{m}^{\left(\mathrm{II}\right)}$ are found for, respectively, gratings I and II (figure 2).

Setting the displacement *u*(*x*,*y*) to vanish at the rigid pins located at (*ja*,0) and (*s*_1_+*la*,*s*_2_), we obtain a system of linear algebraic equations for the coefficients $A_{n}^{\left(I\right)}$ and $A_{m}^{\left(\mathrm{II}\right)}$,

2.6 $$-e^{i\beta ja\cos\psi }=\sum_{n=0}^{\infty }A_{n}^{\left(I\right)}g(\beta ;ja,0;na,0)+\sum_{m=0}^{\infty }A_{m}^{\left(\mathrm{II}\right)}g(\beta ;ja,0;s_{1}+ma,s_{2}),\;j=0,1,2,\ldots$$

and

2.7 $$\begin{array}{rl} -e^{i\beta [(s_{1}+la)\cos\psi +s_{2}\sin\psi ]} & =\sum_{n=0}^{\infty }A_{n}^{\left(I\right)}g(\beta ;s_{1}+la,s_{2};na,0)\\ & \;+\sum_{m=0}^{\infty }A_{m}^{\left(\mathrm{II}\right)}g(\beta ;s_{1}+la,s_{2};s_{1}+ma,s_{2}),\;l=0,1,2,\ldots . \end{array}$$

This semi-infinite system may be written in the matrix form

2.8 Ga=f,

where **G** is a matrix of the single-source Green’s functions (2.4), **a** is a vector of scattering coefficients $A_{n}^{\left(I\right)},A_{m}^{\left(\mathrm{II}\right)}$ and **f** is a vector of incident wave phases.

The system (2.6)–(2.8) resembles those derived in classical works on the scattering of acoustic waves by semi-infinite gratings [25] and truncated systems [26]. This wave scattering method of solution is employed in §2d for comparison with alternative approaches described in §2b,c.

### (b) The kernel function

For the semi-infinite system of scatterers, one may employ a discrete Wiener–Hopf approach, as first implemented in [25], and recently in a Kirchhoff plate setting in [22–24]. We extend the semi-infinite domain to infinity in the negative direction by introducing the following notations for N,M∈Z:

2.9 $$\begin{array}{rl} u(Na,0) & =\left\{ \begin{array}{ll} 0, & N\geq 0\\ B_{N}^{\left(I\right)}, & N<0 \end{array}\right. \end{array}$$

2.10 $$\begin{array}{rl} u(s_{1}+Ma,s_{2}) & =\left\{ \begin{array}{ll} 0, & M\geq 0\\ B_{M}^{\left(\mathrm{II}\right)}, & M<0 \end{array}\right. \end{array}$$

2.11 $$\begin{array}{rl} \;\text{and}\;u_{\mathrm{inc}}(Na,0) & =F_{N}^{\left(I\right)},\;u_{\mathrm{inc}}(s_{1}+Ma,s_{2})=F_{M}^{\left(\mathrm{II}\right)}. \end{array}$$

Here, $B_{N}^{\left(I\right)}$ and $B_{M}^{\left(\mathrm{II}\right)}$ represent the unknown amplitudes of the total flexural displacement at the points (*Na*,0) and (*s*_1_+*Ma*,*s*_2_) for, respectively, *N*,*M*<0, i.e. in the region to the left of the pair of gratings. The field incident at the array points is denoted by $F_{N}^{\left(I\right)},F_{M}^{\left(\mathrm{II}\right)}$ for all N,M∈Z.

Consider the displacement at two field points **r**_*N*_=(*Na*,0) and **r**_*M*_=(*s*_1_+*Ma*,*s*_2_). Using definitions (2.9)–(2.11), we extend the evaluation of the coefficients $A_{n}^{\left(I\right)},A_{m}^{\left(\mathrm{II}\right)}$ to *m*,*n*<0, setting them to be zero because the pins are not present in this region. Similarly, the notations of $B_{N}^{\left(I\right)}$, $B_{M}^{\left(\mathrm{II}\right)}$ are extended to all N,M∈Z, assuming that $B_{N}^{\left(I\right)}$, $B_{M}^{\left(\mathrm{II}\right)}=0$ for *N*,*M*≥0.

Applying the discrete Fourier transform to equations (2.6), (2.7) and (2.9)–(2.11) and using the Fourier variable *k*, we deduce, respectively,

2.12 $$\begin{array}{rl} \sum_{N=-\infty }^{\infty }B_{N}^{\left(I\right)}\;e^{ikNa} & =\sum_{N=-\infty }^{\infty }F_{N}^{\left(I\right)}\;e^{ikNa}+\sum_{N=-\infty }^{\infty }\sum_{n=-\infty }^{\infty }A_{n}^{\left(I\right)}g(\beta ;Na,0;na,0)\;e^{ikNa}\\ & \;+\sum_{N=-\infty }^{\infty }\sum_{m=-\infty }^{\infty }A_{m}^{\left(\mathrm{II}\right)}g(\beta ;Na,0;s_{1}+ma,s_{2})\;e^{ikNa} \end{array}$$

and

2.13 $$\begin{array}{rl} \sum_{M=-\infty }^{\infty }B_{M}^{\left(\mathrm{II}\right)}\;e^{ikMa} & =\sum_{M=-\infty }^{\infty }F_{M}^{\left(\mathrm{II}\right)}\;e^{ikMa}+\sum_{M=-\infty }^{\infty }\sum_{n=-\infty }^{\infty }A_{n}^{\left(I\right)}g(\beta ;s_{1}+Ma,s_{2};na,0)\;e^{ikMa}\\ & \;+\sum_{M=-\infty }^{\infty }\sum_{m=-\infty }^{\infty }A_{m}^{\left(\mathrm{II}\right)}g(\beta ;s_{1}+Ma,s_{2};s_{1}+ma,s_{2})\;e^{ikMa}. \end{array}$$

By a change of indices of summation, the above equations can be rewritten in the form

2.14 $$\begin{array}{rl} \sum_{N=-\infty }^{\infty }B_{N}^{\left(I\right)}\;e^{ikNa} & =\sum_{N=-\infty }^{\infty }F_{N}^{\left(I\right)}\;e^{ikNa}+\sum_{n=-\infty }^{\infty }A_{n}^{\left(I\right)}\;e^{ikna}\sum_{j=-\infty }^{\infty }g(\beta ;ja,0;0,0)\;e^{ikja}\\ & \;+\sum_{m=-\infty }^{\infty }A_{m}^{\left(\mathrm{II}\right)}\;e^{ikma}\sum_{j=-\infty }^{\infty }g(\beta ;ja,0;s_{1},s_{2})\;e^{ikja} \end{array}$$

and

2.15 $$\begin{array}{rl} \sum_{M=-\infty }^{\infty }B_{M}^{\left(\mathrm{II}\right)}\;e^{ikMa} & =\sum_{M=-\infty }^{\infty }F_{M}^{\left(\mathrm{II}\right)}\;e^{ikMa}+\sum_{n=-\infty }^{\infty }A_{n}^{\left(I\right)}\;e^{ikna}\sum_{j=-\infty }^{\infty }g(\beta ;s_{1}+ja,s_{2};0,0)\;e^{ikja}\\ & \;+\sum_{m=-\infty }^{\infty }A_{m}^{\left(\mathrm{II}\right)}\;e^{ikma}\sum_{j=-\infty }^{\infty }g(\beta ;s_{1}+ja,s_{2};s_{1},s_{2})\;e^{ikja}. \end{array}$$

Here, we adopt the notation of [22–24],

2.16 $${\hat{B}}_{-}^{\left(\alpha \right)}=\sum_{N=-\infty }^{\infty }B_{N}^{\left(\alpha \right)}\;e^{ikNa},\;{\hat{F}}^{\left(\alpha \right)}=\sum_{N=-\infty }^{\infty }F_{N}^{\left(\alpha \right)}\;e^{ikNa},\;{\hat{A}}_{+}^{\left(\alpha \right)}=\sum_{n=-\infty }^{\infty }A_{n}^{\left(\alpha \right)}\;e^{ikna},\;\alpha =\text{I},\text{II}.$$

Using the quasi-periodicity of the gratings, we define the grating Green’s function by

2.17 $$\hat{G}(\beta ,k;\xi^{\left(1\right)};\xi^{\left(2\right)})=\sum_{j=-\infty }^{\infty }g(\beta ;ja{\text{e}}_{\text{1}}+\xi^{\left(1\right)};\xi^{\left(2\right)})\;e^{ikja}.$$

We see from (2.14) and (2.15) that the only choices of ***ξ***^(1)^ and ***ξ***^(2)^ that are required are the zero vector and **s**, which identify the front pins of the two shifted gratings. The additional summation over the coefficients $A_{n}^{\left(\alpha \right)}$ in (2.14), (2.15) is then applied and we obtain the functional equation

2.18 $$\left(\begin{array}{c} {\hat{B}}_{-}^{\left(I\right)}\\ {\hat{B}}_{-}^{\left(\mathrm{II}\right)} \end{array}\right)=\left(\begin{array}{cc} \hat{G}(\beta ,k;\text{0};\text{0}) & \hat{G}(\beta ,k;\text{0};\text{s})\\ \hat{G}(\beta ,k;\text{s};\text{0}) & \hat{G}(\beta ,k;\text{s};\text{s}) \end{array}\right)\left(\begin{array}{c} {\hat{A}}_{+}^{\left(I\right)}\\ {\hat{A}}_{+}^{\left(\mathrm{II}\right)} \end{array}\right)+\left(\begin{array}{c} {\hat{F}}^{\left(I\right)}\\ {\hat{F}}^{\left(\mathrm{II}\right)} \end{array}\right),$$

whose kernel function is a matrix of the grating Green’s functions G. With reference to (2.17) and the symmetry relations

2.19 $$\hat{G}(\beta ,k;\text{0};\text{s})=\hat{G}(\beta ,k;-\text{s};\text{0})\;\text{and}\;\hat{G}(\beta ,k;\text{s};\text{s})=\hat{G}(\beta ,k;\text{0};\text{0}),$$

the system (2.18) can be rewritten so that all the elements are referenced to the origin (***ξ***^(2)^=**0**):

2.20 $$\left(\begin{array}{c} {\hat{B}}_{-}^{\left(I\right)}\\ {\hat{B}}_{-}^{\left(\mathrm{II}\right)} \end{array}\right)=\left(\begin{array}{cc} \hat{G}(\beta ,k;\text{0};\text{0}) & \hat{G}(\beta ,k;-\text{s};\text{0})\\ \hat{G}(\beta ,k;\text{s};\text{0}) & \hat{G}(\beta ,k;\text{0};\text{0}) \end{array}\right)\left(\begin{array}{c} {\hat{A}}_{+}^{\left(I\right)}\\ {\hat{A}}_{+}^{\left(\mathrm{II}\right)} \end{array}\right)+\left(\begin{array}{c} {\hat{F}}^{\left(I\right)}\\ {\hat{F}}^{\left(\mathrm{II}\right)} \end{array}\right).$$

Equations (2.18) and (2.20) are of the form

2.21 $${\text{b}}_{-}=G\alpha_{+}+\gamma ,$$

with the vectors **b**_−_, ***α***_+_, ***γ*** representative of scattering coefficients for *x*<0, *x*≥0 and the incident field, respectively.

Multiple gratings extend the case of the scalar Wiener–Hopf equation, analysed in [22,24], to the matrix form. A thorough study of the interaction of a time-harmonic plane wave with a semi-infinite lattice of identical circular cylinders was provided in [32,33], whereby the assumption that finite-sized cylinders do not scatter isotropically led to a matrix Wiener–Hopf equation. Tymis & Thompson [33] adopted a method using the truncation of multipole expansions to derive an approximate system, solved by matching poles and residues on opposing sides. In this way, the necessity to factorize the matrix kernel was avoided.

Factorization of the matrix kernel G is also not required in our approach. As discussed in [22–24], an important feature of the discrete Wiener–Hopf method for semi-infinite platonic crystals is the direct connection between the kernel function and the quasi-periodic Green’s functions for analogous infinite systems. Zeros of these functions correspond to Bloch modes, meaning that analysis of the determinant of G provides information to identify special frequency regimes that support transmission and reflection effects in semi-infinite grating pairs.

We refer to [34], where the kernel function of the Wiener–Hopf equation was used to analyse the properties of waves in a structured medium. Specifically in ch. 11 of [34], Slepyan evaluated the global-to-local energy release ratio associated with the advancing crack without solving the Wiener–Hopf equation, instead using only its kernel function. In a similar way, we use the kernel G of the Wiener–Hopf formulation above in order to make a formal connection with the grating Green’s function. This includes essential information about the waveguide modes and blockages of waves, which is discussed further in subsequent sections.

We consider the case where the pair of semi-infinite gratings are close to one another. The off-diagonal entries $\hat{G}(\beta ,k;\xi^{\left(1\right)};\xi^{\left(2\right)})$ are approximated by expanding about the origin for |***ξ***|≪1. The derivation of these representations, and illustrative examples demonstrating the efficiency of the approximation, are presented in the electronic supplementary material, appendix A. In the next section, we present the related concept whereby the pair of closely spaced pinned gratings is replaced by a single line of point inclusions to which we assign a source and a dipole.

### (c) Single semi-infinite line of sources and dipoles

For the case of closely spaced gratings in figure 2 (|**s**|≪1), the problem may be converted to that for a single array of scatterers, each of which exhibits monopole- and dipole-scattering responses.

#### (i) Notations and definitions

The scattered field may be approximated in the form

2.22 $$u_{\mathrm{sc}}(\xi )≃Sg(\beta ;\xi ;\xi^{d})+D\text{q}\cdot \nabla_{\xi^{d}}g(\beta ;\xi ;\xi^{d}),$$

where **q** is a unit vector. The first term in (2.22) is referred to as the *source* term at ***ξ***^*d*^, and its coefficient *S* is the *source strength*. The second term in (2.22) is referred to as the *dipole* term at ***ξ***^*d*^, and D is the *dipole coefficient*. We note that **q** characterizes the orientation of the dipole; the angle between the direction of **q** and the direction of the positive *x*-axis is referred to as the dipole angle *θ*, and is prescribed in the interval 0≤*θ*≤*π*. The approximation discussed here employs a displacement field ansatz using a Green’s function expansion.

Consider the simple case of a pair of pins positioned at, respectively, ***ξ***^(1)^ and ***ξ***^(2)^. The scattered displacement field *u*_sc_ may be expressed in the form

2.23 $$u_{\mathrm{sc}}(\xi )=A_{1}g(\beta ;\xi ;\xi^{\left(1\right)})+A_{2}g(\beta ;\xi ;\xi^{\left(2\right)}),$$

where *A*_1_,*A*_2_ are scattering coefficients, *g* is the single-source Green’s function (2.4) and ***ξ***=(*x*,*y*) is a general field point. This representation can be rewritten as

2.24 $$\begin{array}{r} u_{\mathrm{sc}}(\xi )=\frac{1}{2}(A_{1}+A_{2})[g(\beta ;\xi ;\xi^{\left(1\right)})+g(\beta ;\xi ;\xi^{\left(2\right)})]+\frac{1}{2}(A_{2}-A_{1})[g(\beta ;\xi ;\xi^{\left(2\right)})-g(\beta ;\xi ;\xi^{\left(1\right)})]. \end{array}$$

Consider the case when |***ξ***^(2)^−***ξ***^(1)^|≪1, and ***ξ*** is fixed. Taking ***ξ***^(2)^=***ξ***^(1)^+**s**,|**s**|≪1,

2.25 $$g(\beta ;\xi ;\xi^{\left(1\right)}+\text{s})-g(\beta ;\xi ;\xi^{\left(1\right)})=\text{s}\cdot \nabla_{\xi^{\left(1\right)}}g(\beta ;\xi ;\xi^{\left(1\right)})+O(|\text{s}{|}^{2}).$$

Then the approximation to the scattered field is

2.26 $$\begin{array}{rl} u_{\mathrm{sc}}(\xi ) & ≃\frac{1}{2}(A_{1}+A_{2})[2g(\beta ;\xi ;\xi^{\left(1\right)})+\text{s}\cdot \nabla_{\xi^{\left(1\right)}}g(\beta ;\xi ;\xi^{\left(1\right)})]+\frac{1}{2}(A_{2}-A_{1})\text{s}\cdot \nabla_{\xi^{\left(1\right)}}g(\beta ;\xi ;\xi^{\left(1\right)})\\ & ≃(A_{1}+A_{2})g(\beta ;\xi ;\xi^{\left(1\right)})+A_{2}\;\text{s}\cdot \nabla_{\xi^{\left(1\right)}}g(\beta ;\xi ;\xi^{\left(1\right)}). \end{array}$$

**Remark.**

Alternatively, one may expand about the vector ***ξ***^*d*^, halfway between the two sources

2.27 $$\xi^{d}=\xi^{\left(1\right)}+\frac{1}{2}\text{s}\;\mathrm{and}\;\xi^{d}=\xi^{\left(2\right)}-\frac{1}{2}\text{s}.$$

Substituting these expressions into (2.24), we obtain

2.28 $$u_{\mathrm{sc}}(\xi )≃(A_{1}+A_{2})g(\beta ;\xi ;\xi^{d})+\frac{A_{2}-A_{1}}{2}\;\text{s}\cdot \nabla_{\xi^{d}}g(\beta ;\xi ;\xi^{d}).$$

We note that this representation is characteristic of a load comprising the sum *A*_1_+*A*_2_ for the source strength, and the difference *A*_2_−*A*_1_ for the dipole coefficient. However, for the subsequent derivations and illustrative examples, we use the representation (2.26), where the approximation is determined by expanding about the point ***ξ***^(1)^. These expressions contain the familiar *A*_1_+*A*_2_ form for the source strengths, but the dipole coefficient is replaced by *A*_2_|**s**|.

#### (ii) Dipole approximation to replace two semi-infinite rows of pins

We consider the alternative formulation for the problem described in §2b, whereby the two arrays of pins are replaced by one semi-infinite line, and both a source strength *S*_*n*_ and a dipole coefficient *D*_*n*_ are associated with each member of the array. We note that, for the sake of convenience, the coefficients *D*_*n*_ are combined with the non-unit shift vector **s** (i.e. for $D_{n}$ of (2.22), $|\text{s}|D_{n}=D_{n}$), but the term ‘dipole coefficients’ will be used for *D*_*n*_ in what follows. We impose two conditions on the total displacement *u*(*x*,*y*) defined at each point ***ξ***=(*ja*,0)

2.29 $$u(ja,0)=0;\;\text{s}\cdot \nabla u{|}_{\xi =(ja,0)}=\frac{\partial u}{\partial \text{s}}(ja,0)=0,$$

which physically implies that the source and the dipole are both located at the point ***ξ***=(*ja*,0).

Recalling equations (2.5) and (2.26), we write the approximation to the total displacement field at a point ***ξ*** in the form

2.30 $$u(\xi )≃u_{\mathrm{inc}}(\xi )+\sum_{n=0}^{\infty }[S_{n}g(\beta ;\xi ;\xi^{\left(1\right)})+D_{n}\text{s}\cdot \nabla_{\xi^{\left(1\right)}}g(\beta ;\xi ;\xi^{\left(1\right)})]{|}_{\xi^{\left(1\right)}=(na,0)}.$$

Consider ***ξ***=(*ja*,0), *j*=0,1,2,…. Referring to equations (2.6), (2.7), we have a system of linear algebraic equations for the coefficients *S*_*n*_ and *D*_*n*_,

2.31 $$-e^{i\beta ja\cos\psi }=\sum_{n=0}^{\infty }[S_{n}g(\beta ;ja,0;na,0)+D_{n}\text{s}\cdot \nabla_{\xi^{\left(1\right)}}g(\beta ;ja,0;na,0)],\;j=0,1,2,\ldots$$

and

2.32 $$\begin{array}{rl} -\text{s}\cdot \nabla_{\xi }\;e^{i\beta \xi \cdot (\cos\psi ,\sin\psi )}{|}_{\xi =(ja,0)} & =\sum_{n=0}^{\infty }[S_{n}\;\text{s}\cdot \nabla_{\xi }g(\beta ;ja,0;na,0)\\ & \;+D_{n}\;\text{s}\cdot \nabla_{\xi }(\text{s}\cdot \nabla_{\xi^{\left(1\right)}}g(\beta ;ja,0;na,0))],\;j=0,1,2,\ldots . \end{array}$$

The notation for the directional derivatives distinguishes between differentiating with respect to the first and second arguments, ***ξ***=(*ja*,0) and ***ξ***^(1)^=(*na*,0). The details required to evaluate the coefficients *S*_*n*_, *D*_*n*_ using the wave scattering method are presented in the electronic supplementary material, appendix B; see equations (B.5), (B.6).

Following the discrete Wiener–Hopf derivation of §2b, we recall the notation of (2.9)–(2.11) for the unknown displacement amplitudes in the ‘reflection’ region for *n*<0, and for the incident plane wave coefficients. Consider a specific point ***ξ***=(*Na*,0) and extend the definitions of *S*_*n*_ and *D*_*n*_ for *n*<0. Applying the discrete Fourier transform to (2.31), (2.32), as in §2b, we derive the algebraic system of equations for the Wiener–Hopf formulation for the dipole approximation:

2.33 $$\begin{array}{rl} \sum_{N=-\infty }^{\infty }B_{N}\;e^{ikNa} & =\sum_{N=-\infty }^{\infty }F_{N}\;e^{ikNa}\\ & \;+\sum_{n=-\infty }^{\infty }\;e^{ikna}(S_{n}+D_{n}\;\text{s}\cdot \nabla_{\xi^{\left(1\right)}})\left(\sum_{j=-\infty }^{\infty }g(\beta ;ja,0;0,0)\;e^{ikja}\right) \end{array}$$

and

2.34 $$\begin{array}{rl} \sum_{N=-\infty }^{\infty }B_{N}^{\prime }\;e^{ikNa} & =\sum_{N=-\infty }^{\infty }F_{N}^{\prime }\;e^{ikNa}\\ & \;+\sum_{n=-\infty }^{\infty }\;e^{ikna}\;\text{s}\cdot \nabla_{\xi }(S_{n}+D_{n}\;\text{s}\cdot \nabla_{\xi^{\left(1\right)}})\left(\sum_{j=-\infty }^{\infty }g(\beta ;ja,0;0,0)\;e^{ikja}\right), \end{array}$$

where we note that now ***ξ***=(*ja*,0) and ***ξ***^(1)^=(0,0). Here we have the following definitions for $B_{N},B_{N}^{\prime },F_{N},F_{N}^{\prime }$ for N∈Z:

2.35 $$\begin{array}{rl} u(Na,0) & =\left\{ \begin{array}{ll} 0, & N\geq 0\\ B_{N}, & N<0 \end{array}\right. \end{array}$$

2.36 $$\begin{array}{rl} \text{s}\cdot \nabla_{\xi }u{|}_{\left(Na,0\right)} & =\left\{ \begin{array}{ll} 0, & N\geq 0\\ B_{N}^{\prime }, & N<0 \end{array}\right. \end{array}$$

2.37 $$\begin{array}{rl} \;\text{and}\;u_{\mathrm{inc}}(Na,0) & =F_{N},\;-\text{s}\cdot \nabla_{\xi }u_{\mathrm{inc}}{|}_{\left(Na,0\right)}=-i\beta (s_{1}\cos\psi +s_{2}\sin\psi )\;e^{i\beta \cos\psi Na}=F_{N}^{\prime }. \end{array}$$

We note that $B_{N}=0,B_{N}^{\prime }=0$ for *N*≥0. We observe that, in this Wiener–Hopf formulation for the dipole approximation, we employ only the quasi-periodic Green’s function (2.17) defining a single array of points $\hat{G}(\beta ,k;\text{0};\text{0})$ in contrast with the full system (2.20), where the kernel matrix includes three grating Green’s functions, shifted relative to one another.

The grating Green’s function $\hat{G}(\beta ,k;\text{0};\text{0})$ is an important and well-studied object in the analysis of platonic grating systems, and has been used numerous times in the literature; see, for example, [13–20]. Refined accelerated convergence formulae have been derived [18], and are implemented here in finding the zeros of the determinants of the kernel matrices for the corresponding semi-infinite problems. We note that alternative methods for evaluating the lattice and grating Green’s functions in pinned plates have been discussed in [13,14,24,35], among others.

The discrete Wiener–Hopf derivation described above, and its kernel in particular, provides a highly efficient procedure for determining the ranges of frequency for scattering effects and trapped waveforms. The motivation for formulating the Wiener–Hopf problem is not to find its explicit solution but to use the connection between its kernel and quasi-periodic Green’s functions, the zeros of which give us the frequencies and incident angles to demonstrate standing waves and localization in the semi-infinite grating systems.

### (d) Illustrative examples

In this section, we demonstrate the efficacy of the dipole approximation by comparing both scattering coefficients and the resulting displacement field plots for the approaches described in §2b,c. Referring to equation (2.30), the dipole approximation yields *S*_*n*_ (source strengths) and *D*_*n*_ (dipole coefficients), where *n* denotes the pin number. The direct approach for a pair of semi-infinite gratings in §2b determines coefficients $A_{n}^{\left(I\right)}$ (for the lower grating) and $A_{n}^{\left(\mathrm{II}\right)}$ (for the upper grating).

By analogy with equation (2.26), the coefficients *S*_*n*_, *D*_*n*_ may be associated with the coefficient terms $A_{n}^{\left(I\right)}+A_{n}^{\left(\mathrm{II}\right)}$ and $A_{n}^{\left(\mathrm{II}\right)}$, respectively. Here we include several numerical examples, with a selection of graphical plots of coefficients for a range of parameter settings, noting that periodicity/spacing *a* is set to unity. The method for solving the system (2.31), (2.32) for a truncated semi-infinite system is outlined in detail in the electronic supplementary material, appendix B, where the truncation parameter *L* indicates the number of points in the array. The analysis involves the use of an asymptotic term for the logarithmic singularity arising for the second derivative terms in (2.32) when *j*=*n*.

#### (i) Scattering and transmission resonance

**Figure 3.**
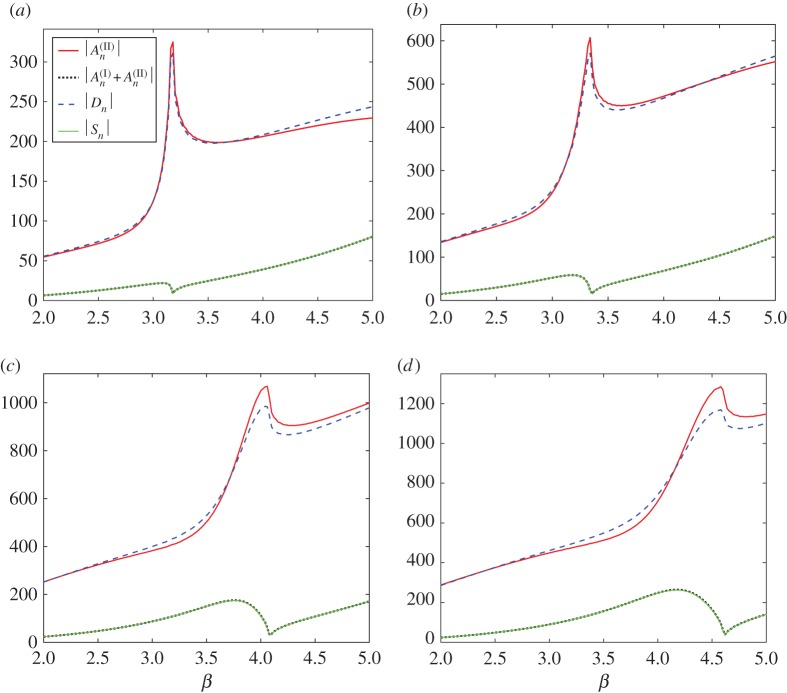
Comparison of the moduli of scattering coefficients evaluated using the wave scattering method (2.6)–(2.8) for a pair of shifted gratings (source terms indicated by dotted lower curves and dipole terms by solid upper curves; see key) and a single semi-infinite line (2.31) and (2.32) of sources (|*S*_*n*_|, solid lower curves) and dipoles (|*D*_*n*_|, dashed upper curves). For shift vector **s**= (0.005,0.015) with |**s**|=0.0158, *θ*=1.25, *L*=160, we consider four angles of incidence: (*a*) *ψ*=0.2, (*b*) *ψ*=0.5, (*c*) *ψ*=1.0, (*d*) *ψ*=1.2. (Online version in colour.)

In figure 3, we plot curves of the moduli for all four sets of the coefficients, two from each of the formulations, for truncation parameter *L*=160. The shifted pair coefficients $|A_{n}^{\left(\mathrm{II}\right)}|$ and $|A_{n}^{\left(I\right)}+A_{n}^{\left(\mathrm{II}\right)}|$ are plotted with solid (upper) curves and dotted (lower) curves, respectively (see the key within the figure). The dipole coefficients |*D*_*n*_| and source strengths |*S*_*n*_| are illustrated by dashed (upper) curves and solid (lower) curves. For a fixed shift vector **s**=(0.005,0.015), with |**s**|=0.0158 and dipole angle *θ*=arccot(*s*_1_/*s*_2_)=1.25, we consider four angles of incidence in radians: *ψ*=0.2,0.5,1.0 and 1.2. The coefficients are plotted for *β* in the range 2≤*β*≤5.

The agreement for the coefficients representing sources (that is, *S*_*n*_ and $A_{n}^{\left(I\right)}+A_{n}^{\left(\mathrm{II}\right)}$) is excellent for all cases as shown in figure 3, where the two curves are visually indistinguishable in each of parts (*a*)–(*d*). There is also very good agreement between |*D*_*n*_| and $|A_{n}^{\left(\mathrm{II}\right)}|$ for all choices of *ψ*. We note that, as *ψ* increases from figure 3*a* to figure 3*d*, the moduli of the dipole coefficients increase, and that the shape of the curve is qualitatively the same for all values of *ψ* considered. Note that the typical sharp peak in the dipole coefficient |*D*_*n*_| (or $|A_{n}^{\left(\mathrm{II}\right)}|$ for the pair) occurs for a higher value of *β* as *ψ* is increased, and its frequency is in the neighbourhood of that of the dip for the source strength curves.

**Figure 4.**
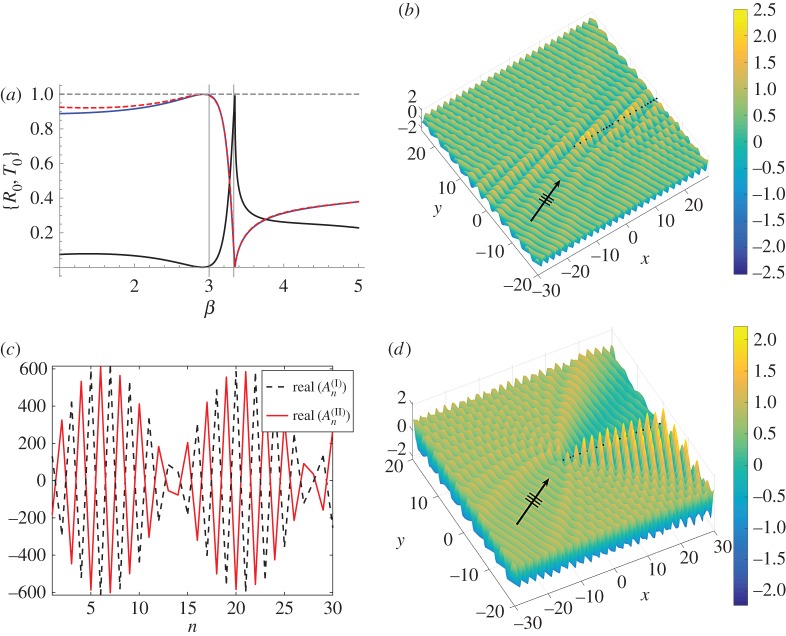
Plane wave with *ψ*=0.5 incident on a pair of gratings with *a*=1.0, **s**=(0.005,0.015). (*a*) Normalized reflected energy for the zeroth propagating order versus *β* for a single line of pins (solid curve with starting value of *R*_0_≈0.9) and a pair of shifted infinite gratings defined by **s** (dashed), and normalized transmitted energy for the pair (solid). Semi-infinite system (*b*) real part of the total displacement field for the first 30 pairs of pins for *β*=3.33, *L*=100. (*c*) Real parts of the scattering coefficients for the first 30 pins. (*d*) Real part of the total displacement field for *β*=3.0; all other parameters are the same as in (*b*). (Online version in colour.)

In this platonic setting, the sharp peaks are associated with additional spectral orders becoming propagating (rather than evanescent), and are linked to transmission resonances. For this example of |**s**|=0.0158, the dipole approximation appears to be robust since the singularity is well approximated for both formulations. We illustrate an example in figure 4 for one of the incident angles *ψ*=0.5 for the spectral parameter *β*=3.33, which is just below the peak frequency arising for *β*=3.35 in figure 3*b*.

In figure 4*a*, we illustrate the transmission resonance using energy plots for the zeroth order for both an infinite single grating and a pair of shifted gratings (defined by **s**=(0.005,0.015)). Normalized reflected (*R*_0_) and transmitted (*T*_0_) energies for the zeroth order are plotted versus the spectral parameter *β*. The reflected energy for the single grating is shown with the solid curve with an initial value of *R*_0_≈0.9, and the Wood anomaly at *β*≃3.35 signifies the additional order −1 passing from evanescence to propagation. This frequency coincides with both a resonance in transmission (solid curve) and a zero in reflection (dashed curve) for the *pair* for the zeroth order (by the conservation of energy). Note that, for *β*>3.35, the energy for the zeroth order no longer sums to unity owing to the additional contributions (not shown here in figure 4*a*) to the total energy from the new propagating order.

In figure 4*b*,*c*, we show how this resonance for the infinite system is manifested in the semi-infinite system. For **s**=(0.005,0.015), *ψ*=0.5, *β*=3.33, we plot the real part of the total displacement field in figure 4*b* for the first 30 pinned pairs (0≤*n*≤29), with the direction of the incident plane wave indicated by the arrow. The real parts of the corresponding coefficients are shown in figure 4*c*. We observe the transmission resonance associated with the transition of an evanescent to a propagating order. Although scattering effects are present in the vicinity of the leading vertex, there is clear evidence of transmission along, and behind, the grating pair.

In particular, the large amplitudes and envelope function for the scattering coefficients $A_{n}^{\left(\mathrm{II}\right)}$ of figure 4*c* are matched by the displacement field along the line of the first 30 pairs of pins in figure 4*b*. This example coincides with the sharp peaks in both figures 3*b* and 4*a*. The zero in transmission (a reflection mode) for the pair at *β*≃3.0 in figure 4*a* is illustrated for the semi-infinite system in figure 4*d*, which not only exhibits strong reflection but also, on comparison with the field in figure 4*b*, further emphasizes the transmission regime for *β*=3.33.

#### (ii) Magnitude and orientation of dipole

**Figure 5.**
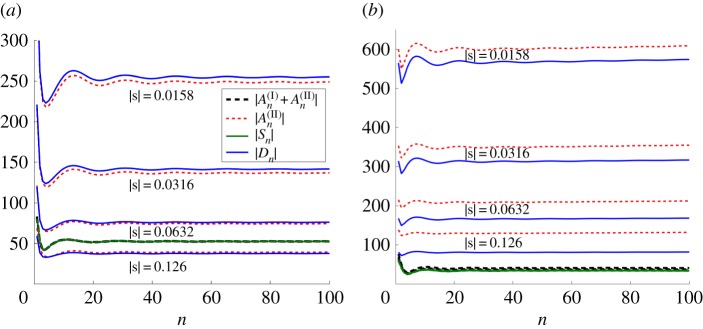
Comparison of scattering coefficients for variation of |**s**| for a pair of gratings ($A_{n}^{\left(I\right)},A_{n}^{\left(\mathrm{II}\right)}$) and for the line of sources and dipoles (*S*_*n*_ and *D*_*n*_). Fixed parameter settings match those of figure 4: ψ=0.5,a=1.0,θ=arctan⁡(3),L=100. (*a*) Reflection frequency of figure 4*d*, *β*=3.0. (*b*) Transmission resonance frequency of figure 4*b*, *β*=3.33. (Online version in colour.)

The dominance of the dipole terms over the source terms for the reflection regime of figure 4*d* is shown in figure 5*a*, where four values of |**s**| (the first of which is |**s**|=0.0158) are shown for the parameter settings of *ψ*=0.5, *β*=3.0. However, as |**s**| is increased, the dipole coefficients tend towards those of the sources. We also include a study of magnitude |**s**| for the transmission case *β*=3.33 in figure 5*b*. Note that, for the resonant frequency, the agreement of *D*_*n*_ and $A_{n}^{\left(\mathrm{II}\right)}$ is reduced.

**Figure 6.**
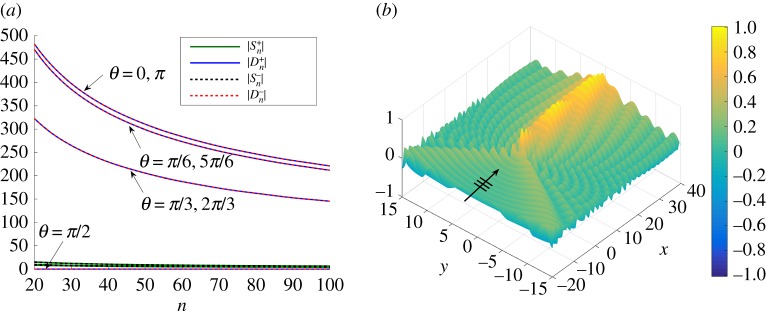
(*a*) Moduli of coefficients for the source and dipole terms for various dipole angles *θ* for normal incidence (*ψ*=0) for upwardly ($S_{n}^{+}$, $D_{n}^{+}$), and downwardly ($S_{n}^{-}$, $D_{n}^{-}$) oriented shifted pairs with |**s**|=0.005, *β*=3.33. The angle *θ* is varied by increments of *π*/6, as indicated by the labels adjacent to the curves of $|D_{n}^{+}|/|D_{n}^{-}|$. (*b*) Scattered field for *θ*=0, |**s**|=0.005, *ψ*=0,*β*=3.33. (Online version in colour.)

Clearly, the magnitude of |**s**| is linked to both the efficiency of the dipole approximation and the relationship between the dipole and source coefficients. A natural question to ask is how does the orientation of the dipole affect the scattering properties of the system? We consider the case of fixing |**s**|=0.005 and varying the dipole angle in the range 0≤*θ*≤*π* (figure 2). Using the same value of *β*=3.33, we investigate the case of normal incidence, *ψ*=0, in figure 6.

We consider two pairs of gratings for each dipole angle *θ*, one oriented upwards, with which we associate the superscript +, and one oriented downwards (effectively defined by −*θ*), with which we associate the superscript −. The moduli of the coefficients for seven choices of *θ*, multiples of *π*/6, are plotted in figure 6*a*. We observe that, in isolation, there is virtually no difference in the results for the upwardly and downwardly oriented shifted pairs. The formulation of a herringbone system by combining these pairs is considered in the next section, including an analysis of the effect of the direction of the dipoles, where naturally the roles of ±*θ* are much more significant.

In figure 6*a*, we note that, as *θ* is increased, the dipole coefficients ($D_{n}^{+}$ and $D_{n}^{-}$) are reduced, and that the dipole coefficients dominate the source strengths with the notable exception of *θ*=*π*/2, which leads to the extreme reduction of all coefficients. This suggests that this orientation of the dipoles for |**s**|≪1, for which the source and dipole coefficients are comparable, replicates the line of equally spaced pins that does not support Rayleigh–Bloch modes [13]. For the parallel direction with *θ*=0, however, some localization is observed, as indicated by the associated scattered field in figure 6*b* for *ψ*=0, |**s**|=0.005.

## 3. Herringbone system of rigid pins

**Figure 7.**
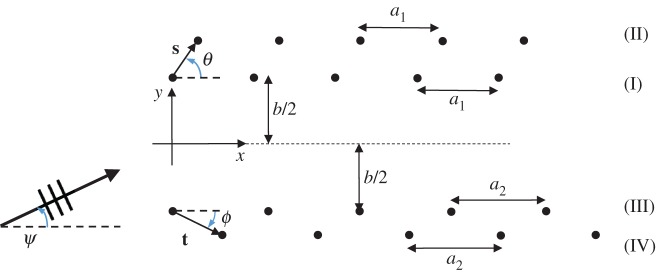
A semi-infinite ‘herringbone’ pattern of of rigid pins in an elastic Kirchhoff plate. The upper pair is characterized by the common spacing *a*_1_ and shift vector **s** (with orientation *θ*), and the lower pair by *a*_2_ and **t** (with orientation *ϕ*). The separation of the pairs of gratings is denoted by *b*. (Online version in colour.)

Consider a herringbone pattern in an elastic Kirchhoff plate, as shown in figure 7, where the upper and lower pairs are characterized by, respectively, the shift vectors **s**=(*s*_1_,*s*_2_) and **t**=(*t*_1_,*t*_2_), and the spacings *a*_1_ and *a*_2_. The separation of the pairs of gratings is denoted by *b*. Note the labelling of the gratings (I)–(IV), which will be used in the examples and their captions that follow.

### (a) Algebraic system

A natural configuration arises for *a*_1_=*a*_2_ and *s*_1_=*t*_1_, *t*_2_=−*s*_2_ for *s*_1_,*s*_2_>0, |**s**|≪1, which may be thought of as a symmetric herringbone with a convex entrance, and a model for a regular double-pinned structure. One may also consider the special cases of *s*_1_<0 (concave entrance) and **t**=−**s**, where the origin of the coordinate axes is shifted such that **s** and **t** both lie on the line *y*=*θx* (wedge).

The formulation of the problem is similar to that outlined in §2. We first consider the general case illustrated by figure 7. The total flexural displacement *u*(*x*,*y*) is given by

3.1 $$\begin{array}{rl} u(x,y) & =u_{\mathrm{inc}}(x,y)+\sum_{n=0}^{\infty }A_{n}^{\left(I\right)}g\left(\beta ;x,y;na_{1},\frac{b}{2}\right)+\sum_{m=0}^{\infty }A_{m}^{\left(\mathrm{II}\right)}g\left(\beta ;x,y;s_{1}+ma_{1},s_{2}+\frac{b}{2}\right)\\ & \;+\sum_{c=0}^{\infty }A_{c}^{\left(\mathrm{III}\right)}g\left(\beta ;x,y;ca_{2},-\frac{b}{2}\right)+\sum_{d=0}^{\infty }A_{d}^{\left(\mathrm{IV}\right)}g\left(\beta ;x,y;t_{1}+da_{2},t_{2}-\frac{b}{2}\right), \end{array}$$

where the scattering coefficients $A_{n}^{\left(I\right)}$, $A_{m}^{\left(\mathrm{II}\right)}$, $A_{c}^{\left(\mathrm{III}\right)}$, $A_{d}^{\left(\mathrm{IV}\right)}$ are to be determined. In a similar way to §2, boundary conditions are applied so that the total displacement *u*(*x*,*y*) vanishes at the rigid pins. We include the details of the method in the electronic supplementary material, appendix C. Here we present only the final results for the case *a*_1_=*a*_2_.

Adopting the notations of (2.16), but for *α*=I–IV, and the shorthand notation from (2.17) together with the additional vector ***τ***=(0,*b*/2), we obtain the functional equation

3.2 $$\left(\begin{array}{c} {\hat{B}}_{-}^{\left(I\right)}\\ {\hat{B}}_{-}^{\left(\mathrm{II}\right)}\\ {\hat{B}}_{-}^{\left(\mathrm{III}\right)}\\ {\hat{B}}_{-}^{\left(\mathrm{IV}\right)} \end{array}\right)=K\left(\begin{array}{c} {\hat{A}}_{+}^{\left(I\right)}\\ {\hat{A}}_{+}^{\left(\mathrm{II}\right)}\\ {\hat{A}}_{+}^{\left(\mathrm{III}\right)}\\ {\hat{A}}_{+}^{\left(\mathrm{IV}\right)} \end{array}\right)+\left(\begin{array}{c} {\hat{F}}^{\left(I\right)}\\ {\hat{F}}^{\left(\mathrm{II}\right)}\\ {\hat{F}}^{\left(\mathrm{III}\right)}\\ {\hat{F}}^{\left(\mathrm{IV}\right)} \end{array}\right),$$

with matrix kernel

3.3 $$K=\left(\begin{array}{cccc} \hat{G}(\beta ,k;\tau ;\tau ) & \hat{G}(\beta ,k;\tau ;\tau +\text{s}) & \hat{G}(\beta ,k;\tau ;-\tau ) & \hat{G}(\beta ,k;\tau ;\text{t}-\tau )\\ \hat{G}(\beta ,k;\tau +\text{s};\tau ) & \hat{G}(\beta ,k;\tau +\text{s};\tau +\text{s}) & \hat{G}(\beta ,k;\tau +\text{s};-\tau ) & \hat{G}(\beta ,k;\tau +\text{s};\text{t}-\tau )\\ \hat{G}(\beta ,k;-\tau ;\tau ) & \hat{G}(\beta ,k;-\tau ;\tau +\text{s}) & \hat{G}(\beta ,k;-\tau ;-\tau ) & \hat{G}(\beta ,k;-\tau ;\text{t}-\tau )\\ \hat{G}(\beta ,k;\text{t}-\tau ;\tau ) & \hat{G}(\beta ,k;\text{t}-\tau ;\tau +\text{s}) & \hat{G}(\beta ,k;\text{t}-\tau ;-\tau ) & \hat{G}(\beta ,k;\text{t}-\tau ;\text{t}-\tau ) \end{array}\right).$$

Note that all the elements are referenced to the origin illustrated in figure 7. Using (2.19), the kernel can be rewritten in the simpler form

3.4 $$K=\left(\begin{array}{cccc} \hat{G}(\beta ,k;\text{0};\text{0}) & \hat{G}(\beta ,k;-\text{s};\text{0}) & \hat{G}(\beta ,k;2\tau ;\text{0}) & \hat{G}(\beta ,k;2\tau -\text{t};\text{0})\\ \hat{G}(\beta ,k;\text{s};\text{0}) & \hat{G}(\beta ,k;\text{0};\text{0}) & \hat{G}(\beta ,k;2\tau +\text{s};\text{0}) & \hat{G}(\beta ,k;2\tau +\text{s}-\text{t};\text{0})\\ \hat{G}(\beta ,k;-2\tau ;\text{0}) & \hat{G}(\beta ,k;-2\tau -\text{s};\text{0}) & \hat{G}(\beta ,k;\text{0};\text{0}) & \hat{G}(\beta ,k;-\text{t};\text{0})\\ \hat{G}(\beta ,k;\text{t}-2\tau ;\text{0}) & \hat{G}(\beta ,k;\text{t}-\text{s}-2\tau ;\text{0}) & \hat{G}(\beta ,k;\text{t};\text{0}) & \hat{G}(\beta ,k;\text{0};\text{0}) \end{array}\right).$$

As previously discussed for the constituent shifted pairs in §2b,c, the zeros of the kernel matrix determine frequency regimes in which the semi-infinite systems may exhibit interesting scattering patterns. The same is true for the herringbone systems described and illustrated here.

### (b) Sources and dipoles

**Figure 8.**
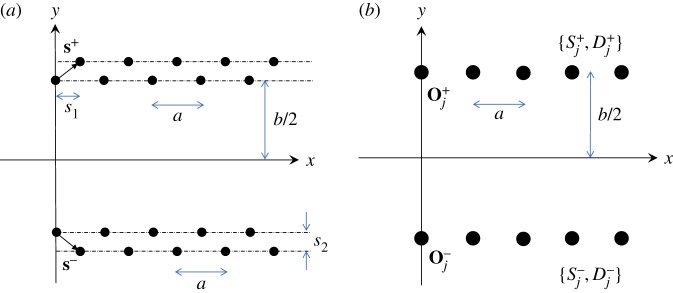
(*a*) Symmetric herringbone system of gratings defined by periodicity *a* and shift vectors **s**^±^. (*b*) The source, dipole approximations. (Online version in colour.)

In a similar way to §2c, we may consider the herringbone system as a pair of semi-infinite lines with both a source strength *S*_*n*_ and a dipole coefficient *D*_*n*_ associated with an individual member of the array. Each pair of gratings is approximated as a single array of point scatterers located at ${\text{O}}_{j}^{\pm }=(ja,\pm b/2)$, as illustrated for the symmetric herringbone with **t**=**s**^−^=(*s*_1_,−*s*_2_) in figure 8. Following the approach defined in equations (2.29) and (2.30), we give the formulation for the herringbone. We begin by imposing the boundary conditions

3.5 $$u{|}_{\text{r}={\text{O}}_{j}^{\pm }}=0,\;{\frac{\partial u}{\partial {\text{s}}^{\pm }}|}_{\text{r}={\text{O}}_{j}^{\pm }}=0,$$

where we employ the expression **s**^+^=**s** for ease of notation. The approximation to the total flexural displacement is expressed as

3.6 $$u(\text{r})≃u_{\mathrm{inc}}(\text{r})+\sum_{\pm }\left[\sum_{j=0}^{\infty }S_{j}^{\pm }g(\beta ;\text{r};{\text{O}}_{j}^{\pm })+\sum_{j=0}^{\infty }D_{j}^{\pm }\frac{\partial g}{\partial {\text{s}}^{\pm }}(\beta ;\text{r};{\text{O}}_{j}^{\pm })\right].$$

Here we associate the coefficients $S_{j}^{\pm }$ with the strengths of the sources, and $D_{j}^{\pm }$ with the coefficients of the dipoles as indicated in figure 8, and $g(\beta ;\text{r};{\text{O}}_{j}^{\pm })$ is the single-source Green’s function as defined by equation (2.4).

Substituting the constraints (3.5) into (3.6), we obtain two systems of equations,

3.7 $$\sum_{\pm }{\left[\sum_{j=0}^{\infty }S_{j}^{\pm }g(\beta ;\text{r};{\text{O}}_{j}^{\pm })+\sum_{j=0}^{\infty }D_{j}^{\pm }\frac{\partial g}{\partial {\text{s}}^{\pm }}(\beta ;\text{r};{\text{O}}_{j}^{\pm })]\right|}_{\text{r}={\text{O}}_{k}^{\pm }}=-u_{\mathrm{inc}}({\text{O}}_{k}^{\pm }),\;k=0,1,2,\ldots$$

and

3.8 $$\sum_{\pm }{\left[\sum_{j=0}^{\infty }S_{j}^{\pm }\frac{\partial g}{\partial {\text{s}}_{\text{r}}}(\beta ;\text{r};{\text{O}}_{j}^{\pm })+\sum_{j=0}^{\infty }D_{j}^{\pm }\frac{\partial }{\partial {\text{s}}_{\text{r}}}\frac{\partial g}{\partial {\text{s}}^{\pm }}(\beta ;\text{r};{\text{O}}_{j}^{\pm })]\right|}_{\text{r}={\text{O}}_{k}^{\pm }}=-\frac{\partial }{\partial {\text{s}}_{\text{r}}}u_{\mathrm{inc}}({\text{O}}_{k}^{\pm }),$$

where

3.9 $$\frac{\partial }{\partial {\text{s}}_{\text{r}}}=\left\{ \begin{array}{ll} \frac{\partial }{\partial {\text{s}}^{+}} & \text{for}\;\text{r}={\text{O}}_{k}^{+},\\ \frac{\partial }{\partial {\text{s}}^{-}} & \text{for}\;\text{r}={\text{O}}_{k}^{-}. \end{array}\right.$$

For the sake of numerical illustrations, we introduce the truncation parameter *L* to represent the number of points in each constituent grating of a restricted system. In that case, we have a 4*L*×4*L* linear algebraic system (3.7)–(3.9) for ${\left\{S_{j}^{\pm },D_{j}^{\pm }\right\}}_{j=0}^{L-1}$.

### (c) Algebraic systems in matrix form for the wave scattering method

The full herringbone system comprising four pinned gratings whose point scatterers enforce zero displacement is defined by (C.2)–(C.5) in the electronic supplementary material. Its matrix form is given by

3.10 $$\left(\begin{array}{c} {\text{F}}^{\left(I\right)}\\ {\text{F}}^{\left(\mathrm{II}\right)}\\ {\text{F}}^{\left(\mathrm{III}\right)}\\ {\text{F}}^{\left(\mathrm{IV}\right)} \end{array}\right)=\left(\begin{array}{cccc} {\text{M}}^{\left(11\right)} & {\text{M}}^{\left(12\right)} & {\text{M}}^{\left(13\right)} & {\text{M}}^{\left(14\right)}\\ {\text{M}}^{\left(21\right)} & {\text{M}}^{\left(11\right)} & {\text{M}}^{\left(23\right)} & {\text{M}}^{\left(24\right)}\\ {\text{M}}^{\left(13\right)} & {\text{M}}^{\left(32\right)} & {\text{M}}^{\left(11\right)} & {\text{M}}^{\left(34\right)}\\ {\text{M}}^{\left(41\right)} & {\text{M}}^{\left(24\right)} & {\text{M}}^{\left(43\right)} & {\text{M}}^{\left(11\right)} \end{array}\right)\left(\begin{array}{c} {\text{A}}^{\left(I\right)}\\ {\text{A}}^{\left(\mathrm{II}\right)}\\ {\text{A}}^{\left(\mathrm{III}\right)}\\ {\text{A}}^{\left(\mathrm{IV}\right)} \end{array}\right),$$

where the various terms **M**^(*ij*)^ are block matrices. For the truncated semi-infinite system with truncation parameter *L*, these blocks are of size *L*×*L* and each of **F**^(*I*)^ to **F**^(*IV*)^ and **A**^(*I*)^ to **A**^(*IV*)^ is an *L*×1 column vector. The 2*L*×2*L* matrix equation for the line array of sources and dipoles approximating a shifted pair is presented in equations (B.5), (B.6) in the electronic supplementary material, appendix B, where the **F** terms incorporate two boundary conditions (2.29) rather than only zero displacement as in (3.10).

For the system (3.7)–(3.9), we derive a similar truncated system where the 4*L*×4*L* matrix may be considered as an array of four 2*L*×2*L* block matrices. The two blocks on the main diagonal are determined using the system (B.5), (B.6), provided that we replace the arguments (*ja*,0) with (*ja*,±*b*/2). Here we shall denote them by $M^{\left(++\right)}({\text{s}}^{+})$ and $M^{\left(--\right)}({\text{s}}^{-})$, where + denotes the upper line and − the lower line (see (3.15) below).

The off-diagonal block matrices take into account the interaction of the upper and lower line arrays, and therefore require expressions that differ from the isolated shifted pair given by (B.3), (B.4). For the first derivative terms in equations (3.7), (3.8), we deduce [36]

3.11 $$\frac{\partial g}{\partial {\text{s}}^{\pm }}(\beta ;\text{r};{\text{O}}_{j}^{\pm })=\frac{i}{8\beta }\left[H_{1}^{\left(1\right)}(\beta \rho_{\xi })+\frac{2i}{\pi }K_{1}(\beta \rho_{\xi })\right]\left(\frac{s_{1}(x-ja)}{\rho_{\xi }}\pm \frac{s_{2}(y\mp b/2)}{\rho_{\xi }}\right),$$

where **r**=(*ka*,∓*b*/2), *k*=0,1,2,… and *ρ*_*ξ*_ is the distance between **r** and ${\text{O}}_{j}^{\pm }$, as defined in (B.1) in the electronic supplementary material. The important difference is that the second group of terms involving *s*_2_ no longer vanish, because *y*=∓*b*/2 is always of opposite sign to the *y*-component of ${\text{O}}_{j}^{\pm }$. Similarly,

3.12 $$\frac{\partial g}{\partial {\text{s}}_{\text{r}}^{\pm }}(\beta ;\text{r};{\text{O}}_{j}^{\pm })=-\frac{i}{8\beta }\left[H_{1}^{\left(1\right)}(\beta \rho_{\xi })+\frac{2i}{\pi }K_{1}(\beta \rho_{\xi })\right]\left(\frac{s_{1}(x-ja)}{\rho_{\xi }}\pm \frac{s_{2}(y\mp b/2)}{\rho_{\xi }}\right).$$

As one would expect, the second derivatives in (3.8) also include more terms:

3.13 $$\begin{array}{rl} \frac{\partial }{\partial {\text{s}}_{\text{r}}}\frac{\partial g}{\partial {\text{s}}^{\pm }}(\beta ;\text{r};{\text{O}}_{j}^{\pm }) & =s_{1}\{\frac{s_{1}g_{\xi }^{\left(1\right)}}{\rho_{\xi }}+\frac{\rho_{\xi }\beta [H_{0}^{\left(1\right)}(\beta \rho_{\xi })-(2i/\pi )K_{0}(\beta \rho_{\xi })]-2g_{\xi }^{\left(1\right)}}{\rho_{\xi }^{3}}\\ & \;\times \left(s_{1}{\left(x-ja\right)}^{2}\pm s_{2}(x-ja)\left(y\mp \frac{b}{2}\right)\right)\}\\ & \;\pm s_{2}\{\pm \frac{s_{2}g_{\xi }^{\left(1\right)}}{\rho_{\xi }}+\frac{\rho_{\xi }\beta [H_{0}^{\left(1\right)}(\beta \rho_{\xi })-(2i/\pi )K_{0}(\beta \rho_{\xi })]-2g_{\xi }^{\left(1\right)}}{\rho_{\xi }^{3}}\\ & \;\times \left(s_{1}(x-ja)\left(y\mp \frac{b}{2}\right)\pm s_{2}{\left(y\mp \frac{b}{2}\right)}^{2}\right)\}, \end{array}$$

where once again *ρ*_*ξ*_ is the distance between **r** and ${\text{O}}_{j}^{\pm }$ and $g_{\xi }^{\left(1\right)}$ is defined as (see also (B.2) in the electronic supplementary material)

3.14 $$g_{\xi }^{\left(1\right)}(\beta ;\text{r};{\text{O}}_{j}^{\pm })=H_{1}^{\left(1\right)}(\beta \rho_{\xi })+\frac{2i}{\pi }K_{1}(\beta \rho_{\xi }).$$

The matrix equation for the system may be expressed in the following way:

3.15 $$\left(\begin{array}{c} F^{+}\\ F^{-} \end{array}\right)=\left(\begin{array}{cc} M^{\left(++\right)}({\text{s}}^{+}) & M^{\left(+-\right)}({\text{s}}^{-})\\ M^{\left(-+\right)}({\text{s}}^{+}) & M^{\left(--\right)}({\text{s}}^{-}) \end{array}\right)\left(\begin{array}{c} T^{+}\\ T^{-} \end{array}\right),$$

where the two 2*L*×2*L* block matrices $M^{\left(-+\right)}$ and $M^{\left(+-\right)}$ give the information regarding the interaction of the upper array (governed by the shift vector **s**^+^) and the lower array (characterized by **s**^−^), evaluated using equations (3.11)–(3.14). The two column vectors have size 4*L*×1, consisting of two concatenated 2*L*×1 vectors defined by

3.16 $$F^{+}=\left(\begin{array}{c} {\text{F}}_{1}^{+}\\ {\text{F}}_{2}^{+} \end{array}\right),\;F^{-}=\left(\begin{array}{c} {\text{F}}_{1}^{-}\\ {\text{F}}_{2}^{-} \end{array}\right);\;T^{+}=\left(\begin{array}{c} {\text{S}}^{+}\\ {\text{D}}^{+} \end{array}\right)\;\mathrm{and}\;T^{-}=\left(\begin{array}{c} {\text{S}}^{-}\\ {\text{D}}^{-} \end{array}\right).$$

The source and dipole coefficients for the upper and lower arrays in figure 8, $S_{j}^{\pm },D_{j}^{\pm }$, are represented by the column vectors on the right-hand side of (3.15). The column vectors ${\text{F}}_{1}^{\pm }$ and ${\text{F}}_{2}^{\pm }$ represent the two sets of boundary conditions on the right-hand sides of (3.7), (3.8) for each of the corresponding line arrays. In the illustrative examples that follow, both systems (3.10) and (3.15) are solved and compared.

### (d) Herringbone systems for waveguiding and localization

We recall the work of Jones *et al.* [24], who used the connection between the channelling of trapped modes in a pair of semi-infinite gratings and the Bloch–Floquet analysis for the infinite waveguide. The Bloch modes are obtained by solving the eigenvalue problem for the matrix of governing grating Green’s functions, which is equivalent to finding the zeros of the determinant of the kernel matrix of Green’s functions presented in [24] and here. Considering the case of normal incidence, the authors presented a contour plot in fig. 3(b) of [24] that identifies the range of values of the spectral parameter *β* and grating separation *b* to support waveguide modes. An effective waveguide model for the simply supported boundary condition was derived, which can be used in conjunction with the Bloch–Floquet analysis to estimate the wavenumber *k*_*x*_, and hence wavelength λ_w_, for the first-order waveguide modes

3.17 $$b=\frac{\pi }{\sqrt{\beta^{2}-k_{x}^{2}}},\;\lambda_{w}=\frac{2\pi }{k_{x}}\;\mathrm{and}\;\lambda_{\mathrm{ext}}=\frac{2\pi }{\beta },$$

where the wavelength exterior to the gratings is denoted by λ_ext_.

**Figure 9.**
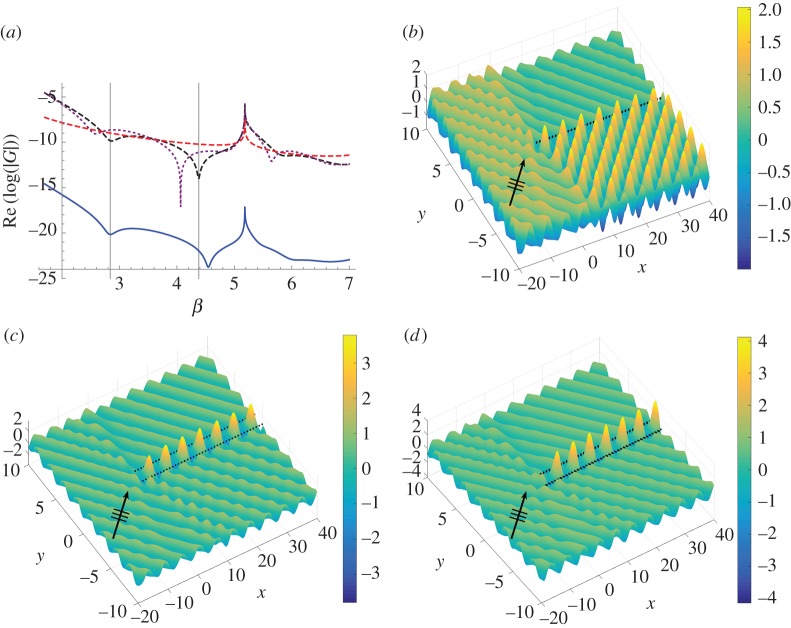
Symmetric herringbone system with $\text{s}=(0.2,0.1),b=\sqrt{2}$, *a*_1_=*a*_2_=1. (*a*) Identification of Bloch modes for the infinite herringbone system for *k*_*x*_=1.1. Solid curve shows eigenmodes for *β*≃2.84,4.55. The upper dashed (referencing the top left corner) curve shows modes for the inner pair (gratings I and III) with spacing $b=\sqrt{2}$, and the lower dashed curve represents the shifted pair (gratings I and II) defined by **s**. The dotted curve shows the outer pair (gratings II and IV) with spacing $\sqrt{2}+0.2$. Parts (*b*–*d*) feature the total displacement field plots for an incident plane wave with *ψ*=1.17 and *β*=2.82 for *L*=100, with the first 40 pins shown. (*b*) Shifted pair (gratings I and II) defined by **s**=(0.2,0.1). (*c*) Inner pair (gratings I and III) separated by $b=\sqrt{2}$. (*d*) Symmetric herringbone (gratings I–IV) defined by the parameters *b* and **s**,**s**^−^. (Online version in colour.)

A similar approach was implemented by Haslinger *et al.* [19] for the connection between the scattering problem and the infinite grating system’s waveguide modes. Using the related eigenvalue problem for a governing matrix of grating Green’s functions dependent on *β* and *k*_*x*_, and incorporating spacing *b*, solutions are obtained in the form of localized minima of the logarithm of the determinant function (see figure 9*a* here, for example). In conjunction with an approximate waveguide model for the Helmholtz operator (neglecting the evanescent modes arising for the biharmonic case), table 1 in [19], and updated here in the electronic supplementary material, appendix D, presents a selection of illustrative parameter settings. For gratings with unit periodicity (*a*=1), various pairs of (*β*,*k*_*x*_) values determine resonant trapped modes for spacings *b* that are well approximated by (3.17).

There is good correspondence between the *β*, *b* pairs in that table and those featured in the white strip illustrating the approximate regions of roots in fig. 3(b) of [24]. The additional feature of table 1 in the electronic supplementary material, appendix D, i.e. providing associated *k*_*x*_ values, may be used to extend this approach from normal to oblique incidence. The projection onto the waveguide’s axis of symmetry,

3.18 $$k_{x}=\beta \;\cos\;\left(\psi \right),\;0\leq \psi \leq \pi ,$$

is used to incorporate oblique angles of incidence *ψ* that correspond to the wavenumbers *k*_*x*_ for normally incident resonant modes in [24]. Both methods provide invaluable insight for the first-order periodic patterns for both the pair and the herringbone systems.

In the examples that follow, the periodicity *a* of all gratings is taken to be unity. We also refer to various constituent pairs of gratings within the herringbone structures. Recalling figure 7, we denote gratings I and III, separated by *b*, as the inner pair. The outer pair refers to gratings II and IV, and a shifted pair is either I, II or III, IV.

#### (i) Waveguide modes

In this section, we describe two examples of waveguide modes. In both cases, we consider a symmetric herringbone system as illustrated in figure 8*a*. In the first case, we show a localized mode that is supported by a simple grating pair waveguide, but which is enhanced, in terms of both amplitude and reduced leakage, by adding the extra gratings to produce the herringbone system. In the second case, the original pair of gratings (I) and (III) reflect a specific range of plane waves, but, by forming the tuned herringbone system, highly localized waveguide modes are observable. In this way, we illustrate how a simple tuning parameter can be used to convert a reflective mode to a highly localized guided waveform.

We consider a symmetric herringbone defined by the parameter choices $\text{s}=(0.2,0.1),b=\sqrt{2}$. The separation $b=\sqrt{2}$ was inspired by Jones *et al*. [24], where the motivation was linked to the presence of Dirac-like points on the dispersion surfaces of doubly periodic systems, as explained in [20]. The Bloch modes of the corresponding infinite herringbone system are obtained by solving the eigenvalue problem for a system of four appropriately positioned pinned gratings. The solutions are illustrated in figure 9*a*, where the appearance of localized minima of the logarithm of the determinant of the system’s governing matrix indicates the presence of Bloch modes. We plot this function versus the spectral parameter *β* in figure 9*a* for *k*_*x*_=1.1.

The solid curve indicates the resonant *β* values for the herringbone structure, the upper dashed (with reference to the top left corner of figure 9*a*) curve for the inner pair (gratings I and III) separated by $b=\sqrt{2}$ and the lower dashed curve for the shifted pair (either I, II or III, IV). For the infinite system, pairs (I), (II) and (III), (IV) are characterized by the same Bloch modes. The outer pair’s modes are shown by the dotted curve (gratings II and IV). We observe that both the herringbone and the inner pair possess two clear modes, whereas the shifted pair possess no modes for this range of frequencies. The first mode occurs for *β*≃2.84 and a line has been added to the figure to indicate the coincident frequency for both the inner pair and the herringbone.

The second vertical line at *β*≃4.38 highlights the location of the second mode for the inner pair, but the herringbone’s second mode arises for a different value, *β*≃4.55. The implication is that, for the higher frequency, the addition of the extra gratings to create the herringbone system induces a resonant mode that would not be apparent for the inner pair at the same frequency. On the contrary, the first mode is seen regardless of the addition of the extra gratings. This analysis is also valid for the semi-infinite herringbone systems, as illustrated in figure 9*b*–*d*, where we consider the case of *β*=2.82 and hence *ψ*=1.17 from (3.18) for *k*_*x*_=1.1. Note that the determinant vanishes at the slightly higher value *β*≃2.84 in figure 9*a*. The infinite system is used to indicate the neighbourhood of values of *β* corresponding to waveguide modes in the semi-infinite structure, and for illustrative purposes we use the truncation parameter *L*=100.

We show the strong reflection for the pair of shifted gratings defined by **s**=(0.2,0.1) in figure 9*b*. The shifted pair (I), (II) exhibits no resonance, which is consistent with the dashed curve in figure 9*a*. However, the inner pair of semi-infinite gratings supports a waveguiding effect consistent with the mode displayed in figure 9*a*. The total displacement field is plotted in figure 9*c*. A regular one-dimensional periodic pattern, with seven clear peaks and wavelength λ_w_=2*π*/*k*_*x*_≃5.71, is observed, shown here for the first 40 pins.

The localized waveguide mode, wherein the incident plane wave (clearly indicated by the arrow in all diagrams) undergoes significant bending to be channelled between the pinned gratings, is observed for the herringbone structure in figure 9*d*. The mode is virtually identical for both cases (*c*) and (*d*), with the same seven regular peaks and same wavelength λ_w_=2*π*/*k*_*x*_≃5.71. However, the addition of the outer gratings reduces the scattering and amplifies the waveguiding localization, as shown in figure 9*d*, thereby enhancing the waveguiding effect. We define an enhancement factor $F_{e}$ as the ratio of peak amplitudes in the central channels of a four-grating herringbone waveguide to that of a two-grating unshifted to that for the same parameter settings,

3.19 $$F_{e}=\frac{|u_{\max}^{\left(I)-(\mathrm{IV}\right)}|}{|u_{\max}^{\left(I),(\mathrm{III}\right)}|}.$$

A value of $F_{e}=1.00$ would indicate that the herringbone system replicates precisely the mode supported by the two-grating waveguide. In the case of figure 9*c*,*d*, the enhancement factor is $F_{e}=1.09$.

By contrast, there is no correspondence for the *β* values of both systems for the second mode in figure 9*a*. The extra shifted gratings alter the frequency of the second Bloch mode that can be excited by appropriately chosen values of *β* and *ψ* in the semi-infinite problem. A larger value of *s*_1_ increases the difference in *β* values for modes trapped by the inner pair and the herringbone structure.

**Figure 10.**
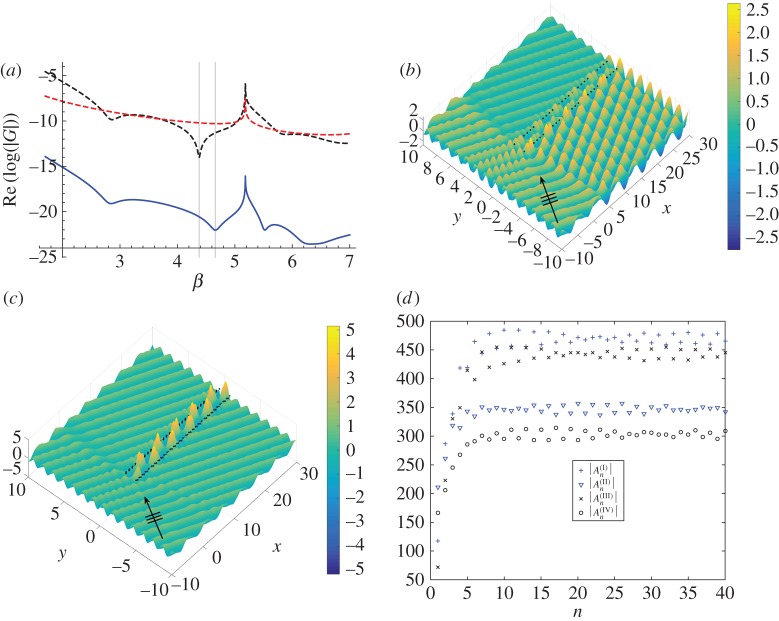
Symmetric herringbone system with $\text{s}=(0.3,0.1),b=\sqrt{2}$. (*a*) Identification of Bloch modes for the infinite herringbone system for *k*_*x*_=1.1. Solid curve shows eigenmodes for *β*≃2.80,4.66,5.53. The upper dashed (referencing the top left corner) curve shows inner pair (gratings I and III) modes, and the lower dashed curve represents the shifted pair (gratings I and II). (*b*,*c*) Total displacement field plots for an incident plane wave with *ψ*=1.3325 and *β*=4.66 for *L*=100 (first 30 shown). (*b*) Inner pair (gratings I and III) with $b=\sqrt{2}$. (*c*) Symmetric herringbone (gratings I–IV) defined by *b* and **s**,**s**^−^. (*d*) Moduli of the scattering coefficients for the herringbone (first 40 shown). (Online version in colour.)

To better illustrate the concept of a pair of gratings that reflect waves at a given frequency being enhanced by an additional pair to support a waveguide transmission, we consider the case of **s**=(0.3,0.1) (increasing *s*_1_ from the value illustrated in figure 9). The logarithm of the determinant of the governing matrix for this system is plotted versus *β* in figure 10*a*. This herringbone system’s second mode occurs for *β*=4.66, compared with that of *β*=4.38 for the inner pair (both marked by vertical lines in figure 10*a*). Thus, the addition of the outer gratings to the inner pair converts a reflection into a waveguide mode.

We show the total displacement field for the inner pair (gratings I and III) for a plane wave incident at *ψ*=1.3325 and *β*=4.66 in figure 10*b*. This choice of *ψ* is determined using equation (3.18) for *β*=4.66 and *k*_*x*_=1.1. Reflection dominates but there is some localization within the grating pair, as can be predicted from the upper dashed curve in figure 10*a*. However, by adding the extra gratings defined by **s**=(0.3,0.1) above, and **s**^−^ below, and exciting the system with the same incident wave, we now observe a highly localized waveguide mode within the herringbone structure (gratings I–IV) in figure 10*c*, with $F_{e}=3.54$.

The moduli of the scattering coefficients for all four gratings are plotted in figure 10*d*, with the first 40 shown. The amplitudes for the inner pair coefficients $A_{n}^{\left(I\right)}$ and $A_{n}^{\left(\mathrm{III}\right)}$ are very similar, which is also true for the first example with *β*=2.82. The important factor here is that they are also of similar order to the outer pair coefficients $A_{n}^{\left(\mathrm{II}\right)}$ and $A_{n}^{\left(\mathrm{IV}\right)}$, which emphasizes that all four gratings are required to support the localized mode, whereas only two gratings were sufficient for the previous case. In this case, the herringbone structure supports a unique waveguide mode, attainable only with the shifted grating structure.

#### (ii) Herringbone systems: dipole terms

The examples of waveguiding demonstrated in figures 9 and 10 arise for, respectively, |**s**|=0.22 and |**s**|=0.32, choices of **s** that are not well approximated using the array of sources and dipoles method outlined in §§2c and 3b. In this section, we consider an example for |**s**|=0.032, where the dipole approximations are found to be valid and the dipole terms dominate the source terms.

**Figure 11.**
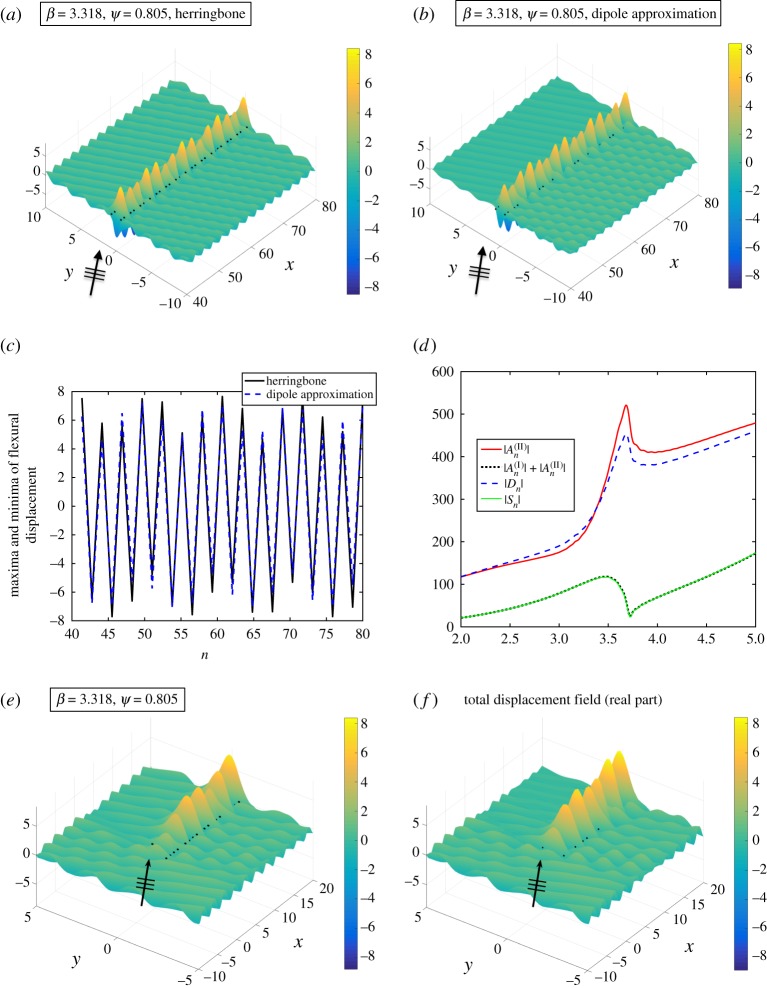
Herringbone system with **s**=(0.01,0.03),*b*=1.3, *a*_1_=*a*_2_=1. Total displacement field plots for an incident plane wave with *ψ*=0.805 and *β*=3.318 for *L*=160 (pins 40–80 shown) for (*a*) a herringbone system comprising four rows of pins. (*b*) Two line arrays of sources and dipoles with coefficients *S*_*n*_ and *D*_*n*_. (*c*) Maxima and minima of the flexural displacements for the herringbone (solid) and two arrays of sources/dipoles (dashed) for the fields in parts (*a*) and (*b*). (*d*) Comparison of coefficients for the upper pair (gratings I and II) of the herringbone system defined by **s**. (*e*,*f*) Comparison of end effects for (*e*) the herringbone and (*f*) two line arrays. (Online version in colour.)

In figure 11*a*, we plot the total displacement field for the herringbone system comprising four rows of pins, with central spacing *b*=1.3 and shift vectors **s**=(0.01,0.03), **t**=(0.01,−0.03), using the truncation parameter *L*=160 (pins 40–80 shown). The incident plane wave is defined by *ψ*=0.805 for *k*_*x*_=2.3 and *β*=3.318 from (3.18). Here the scattering coefficients $A_{n}^{\left(I\right)}$ to $A_{n}^{\left(\mathrm{IV}\right)}$ are determined using the wave scattering method. The bending and waveguiding localization are quite striking, with amplitudes reaching more than eight times those of the incident plane wave. The field shows transmission above the grating system in figure 11*a*, with only very minimal scattering effects evident around the lower pair of gratings, thereby demonstrating the focusing and waveguiding capabilities of a tuned herringbone system.

The relatively small value of |**s**|=0.032 ensures that the source–dipole approach approximates the system well, as illustrated in figure 11*b*–*f*. The displacement field plotted using the source and dipole coefficients $S_{n}^{+}$, $D_{n}^{+}$ for **s**, and those for **s**^−^ of $S_{n}^{-}$, $D_{n}^{-}$ calculated using the system (3.7)–(3.9) described in §3b,c, is shown in figure 11*b* for truncation parameter *L*=160. A very similar localization effect is observed within the system, with the envelope function and location of peaks consistent with figure 11*a*.

Figure 11*c* provides further evidence for investigating the source–dipole approximation approach. The maxima and minima of the flexural displacements are plotted for the fields depicted in parts (*a*) and (*b*), with those of the full herringbone system shown using the solid curve, and those of the dipole approximation shown using the dashed curve. The excellent correspondence is clearly evident, with both the magnitudes and the distribution of the coefficient terms matching well.

The upper pair coefficients, shown in figure 11*d*, where the values for the pair $A_{n}^{\left(I\right)},A_{n}^{\left(\mathrm{II}\right)}$ (solid curve) are compared with the dipole coefficients $D_{n}^{+}$ (dashed curve) for gratings (I) and (II), display excellent correspondence. In particular, there is a very good match in the vicinity of the operating frequency *β*=3.318, where the dashed curve crosses the solid $|A_{n}^{\left(\mathrm{II}\right)}|$ curve.

However, there are some small but discernible differences. As well as the increased reflection visible for −5≤*y*≤0, 40≤*x*≤80 in figure 11*b* compared with figure 11*a*, some of the details of the end effects in the vicinity of the system’s entrance are lost with the dipole approximation, as shown in figure 11*e*,*f*. The full herringbone system (figure 11*e*) has slightly lower amplitudes, and there appears to be a small phase difference when comparing figure 11*f* with figure 11*e*. The small discrepancies are likely to have arisen from the size of |**s**| and this is a resonant example. A non-resonant example for a smaller magnitude of **s**, but the same angle of incidence *ψ* and system parameters *θ*, *b*, is included in the electronic supplementary material, appendix E. In that case, the changes in both the phase and the scattering pattern are significantly reduced.

#### (iii) Dependence on dipole angle

In §2d, and figure 6 in particular, we looked at how scattering patterns depend on the dipole angle. As figure 6 illustrates for normal incidence, the increase of *θ* from being aligned with the incident plane wave to becoming perpendicular reduces the scattering coefficients to zero. In the herringbone system, formed of two arrays of sources and dipoles, the role of the dipole angle *θ* is greatly enhanced. For the same settings (*β*=3.33 and *ψ*=0), we construct a symmetric herringbone system with spacing *b*=1.3 and initial shift vectors **s**=**s**^−^=(0.005,0). We impose the length |**s**|=0.005 to remain constant and vary the angle *θ* in the range 0≤*θ*≤*π*. In this way, the dipole angle for the lower half of the herringbone, denoted by *ϕ* in figure 7, varies in the range 0≥*ϕ*≥−*π*.

**Figure 12.**
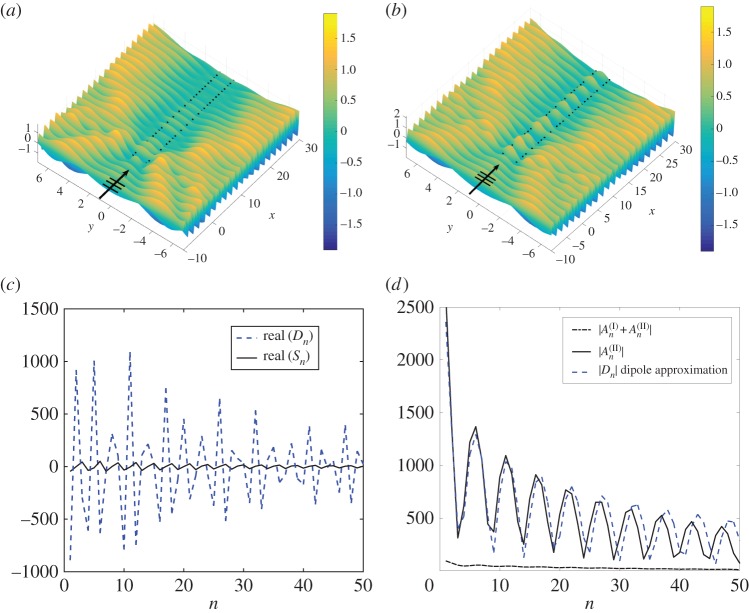
Total displacement fields for *ψ*=0, *β*=3.33, *b*=1.3 for (*a*) a pair of unshifted gratings (gratings I and III), (*b*) the herringbone system with **s**=**t**= (0.005,0), *θ*=0, *L*=100. Real parts of the coefficients are illustrated in (*c*), with dipole (*D*_*n*_, dashed) and source (*S*_*n*_, solid). (*d*) Moduli of scattering coefficients comparing the dipole coefficients for the full herringbone and its approximation. (Online version in colour.)

For the spacing *b*=1.3, frequencies in the neighbourhood of *β*=3.33 can be tuned to support waveguiding effects, as demonstrated by equation (3.17) and in figure 11. An example of blockage for a pair of gratings is obtained for *β*=3.33 and *ψ*=0 in figure 12*a*, where we observe a mode with significantly reduced resolution. The choice of normal incidence is a perfect regime to investigate the design possibilities of varying the dipole angle *θ*, because each array of sources/dipoles is subject to ‘head on’ incidence.

We consider the most basic addition of dipoles first, setting *s*_2_ to be zero and |**s**|=0.005, aligning *θ* with the angle of incidence *ψ*=0. The effect is quite remarkable, with the herringbone system supporting waveguiding and localization in the channel, as shown in figure 12*b* for *L*=100, with the first 30 pins shown. The waveguiding localization is not as impressive as for the tuned design of *ψ*=0.805 and **s**=(0.01,0.03) in figure 11*a*, but the ability to convert a blockage to a leaky waveguide by replacing sources with dipoles is an effect deserving of attention.

In figure 12*c*, the real parts of the source and dipole coefficients are plotted for the first 50 points for the waveguide mode depicted in figure 12*b*. The dominance of the dipole coefficients is clear, and the envelope function of the coefficients is consistent with the displacement field in figure 12*b*. The accuracy of the dipole approximations is also illustrated in figure 12*d*, where the moduli $|A_{n}^{\left(\mathrm{II}\right)}|$ and |*D*_*n*_| are compared. Note once again the relative magnitudes for the source strengths (dotted-dashed curve).

**Figure 13.**
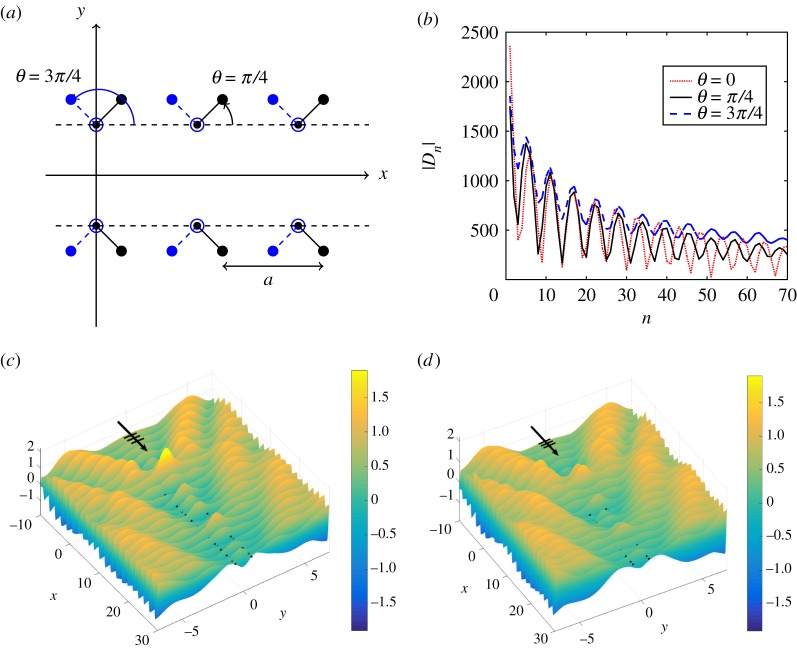
Symmetric herringbone system with |**s**|=0.005,*b*=1.3, **t**=**s**^−^ for normal incidence *ψ*=0 and *β*=3.33, *L*=100. (*a*) Two configurations with *θ*=*π*/4 and *θ*=3*π*/4. (*b*) Comparison of dipole coefficients |*D*_*n*_| for *θ*=0,*π*/4,3*π*/4. (*c*,*d*) Total displacement fields for the herringbone systems with, respectively, *θ*=*π*/4 and *θ*=3*π*/4. (Online version in colour.)

We now consider varying the dipole orientation *θ*. Figure 13*a* shows, schematically, two symmetric waveguides defined by *θ*=*π*/4,3*π*/4. We adopt a consistent classification for the entire figure with the solid lines representing **s**=(0.0035,0.0035) (a convex entrance) and the dashed case being **s**=(−0.0035,0.0035) (the concave entrance). The convexity of the herringbone entrance concomitantly affects the localization, one of the unique features of the model.

The moduli of the dipole coefficients |*D*_*n*_| are plotted in figure 13*b*, with the first 70 points shown (*L*=100). We include those for *θ*=0, discussed in figure 12 and plotted here with a dot-dashed curve, and show that varying *θ* has clear effects on the scattering properties. The greater modulation of the amplitudes for *θ*=0 manifests in a clearly defined waveguide mode in figure 12*b*. The *π*/2 shift in *θ* from *π*/4 to 3*π*/4 has an impact on the coefficients in figure 13*b*, and on the localization in the vicinity of the herringbone entrance. The convex case favours strong localization, as illustrated in figure 13*c*, where the displacement field for *θ*=*π*/4 is plotted, compared with that for *θ*=3*π*/4 in figure 13*d*.

Although the flexural wave fields are similar, there are visual differences in the localization patterns observed within the gratings, with the peaks at the front of the system for *θ*=*π*/4 significantly higher than those for *θ*=3*π*/4. This effect is related to the convexity/concavity of the entrance to the herringbone system. For acute values of *θ*, the symmetric herringbone supports greater localization in the neighbourhood of the opening compared with the case of *π*/2<*θ*<*π*. It is also clear that the choice of a value of *θ* greater than 0 leads to a reduction in the extent of waveguiding through the channel.

## 4. Concluding remarks

In this article, we have presented a new type of flexural waveguide designed in the form of a herringbone system. We have demonstrated that the herringbone system can significantly enhance the localization effects, in terms of amplitudes and focusing, compared with a simpler two-grating waveguide. We have also shown how the herringbone structure is capable of converting a grating pair’s reflection mode into a waveguide mode for the same incident plane wave.

This paper has introduced a novel type of approximation for waveguide modes in structured semi-infinite grating stacks. It is based on the dipole approximation, which takes into account the relative positions and interactions within a structured waveguide such as the herringbone system. This elegant asymptotic approximation is complemented by the derivation of the functional equation of Wiener–Hopf type and analysis of its kernel function.

Several mathematical techniques were implemented, including a classical wave scattering method to derive a system of linear algebraic equations for the flexural displacement. The solution of this system was obtained in the form of scattering coefficients used to plot displacement fields that demonstrate waveguiding effects. A discrete Wiener–Hopf formulation yielded expressions for kernel matrices consisting of quasi-periodic Green’s functions. The zeros of the determinant of such a kernel matrix correspond to Bloch modes for infinite grating systems, the knowledge of which was used to aid the solution of the corresponding semi-infinite scattering problems.

For the case when the shifted pairs consist of two closely spaced gratings, we implemented an alternative mathematical approach. The proximity of pairs of pins advocates the use of a dipole approximation. The validity of the approach for small |**s**| was illustrated with several examples. We showed that both the magnitude and the orientation of the dipoles are important for tuning the localization effects.

We have supplemented the concepts and theoretical analysis with illustrative examples to stimulate further investigations into herringbone waveguides. The model is very rich and we envisage future analysis to quantify the peak amplitudes, enhancement factors, leakage and decay rates, with the possibility of experimental validation. To this end, we anticipate that similar localizing effects will be observed as the radius of the pins becomes finite. For examples of similar extensions to inclusions of finite radii, but for infinite, rather than semi-infinite, platonic gratings, one may consult [37,38]. The infinite herringbone analogue would also be an interesting instrument to analyse transmission resonances, along the lines of the work of [19,39].

## Supplementary Material

Supplementary material for Localisation in semi-infinite herringbone waveguides

## Data accessibility

The work does not include any experimental data. All computational results presented in the examples of the paper are reproducible using the analytical methods detailed in the article and the appendices in the electronic supplementary material.
