# Supplementary material for: Localization in semi-infinite herringbone waveguides
Source: Proc Math Phys Eng Sci. 2018 Mar 28;474(2211):20170590. doi: 10.1098/rspa.2017.0590 (PMC5897755; doi:10.1098/rspa.2017.0590)
Supplement: Supplementary material for Localisation in semi-infinite herringbone waveguides [file rspa20170590supp1.pdf]

PROCEEDINGS A

rspa.royalsocietypublishing.org

Research

Article submitted to journal

Subject Areas:
Applied Mathematics

Keywords:
Flexural waves, platonic crystals, localisation, herringbone systems, Kirchhoff plate.

Author for correspondence:
S.G. Haslinger

Supplementary material for Localisation in semi-infinite herringbone waveguides - Appendices

S.G. Haslinger [1], I.S. Jones [2], N.V. Movchan [1] and A.B. Movchan [1]

[1] Department of Mathematical Sciences, University of Liverpool, Liverpool L69 7ZL, UK
[2] Mechanical Engineering and Materials Research Centre, Liverpool John Moores University, Liverpool L3 3AF, UK

THE ROYAL SOCIETY
PUBLISHING

© The Author(s) Published by the Royal Society. All rights reserved.

A. Dipole approximations for kernel matrix

The structure of the kernel matrix in equation (2.20) suggests that the off-diagonal entries $\hat{G}(\beta, k; \boldsymbol{\xi}^{(1)}; \boldsymbol{\xi}^{(2)})$ can be approximated by expanding about the origin to obtain a dipole-like representation.

For gratings I and II we define, respectively, $\boldsymbol{\xi}^{(1)} = \mathbf{0}$, $\boldsymbol{\xi}^{(2)} = \mathbf{s}$. Then one can say, for $\boldsymbol{\xi}^{(2)} = \boldsymbol{\xi}^{(1)} + \mathbf{s}$, $|\mathbf{s}| \ll 1$,

$$\begin{aligned}
\hat{G}(\beta, k; \boldsymbol{\xi}'^{(1)}, \boldsymbol{\xi}^{(2)}) &= \hat{G}(\beta, k; \boldsymbol{\xi}'^{(1)}; \boldsymbol{\xi}^{(1)} + \mathbf{s}) \simeq \hat{G}(\beta, k; \boldsymbol{\xi}'^{(1)}; \boldsymbol{\xi}^{(1)}) + \mathbf{s} \cdot \nabla \hat{G}(\beta, k; \boldsymbol{\xi}'^{(1)}; \boldsymbol{\xi}^{(1)}), \\
\hat{G}(\beta, k; \boldsymbol{\xi}'^{(2)}; \boldsymbol{\xi}^{(1)}) &= \hat{G}(\beta, k; \boldsymbol{\xi}'^{(1)} + \mathbf{s}; \boldsymbol{\xi}^{(1)}) \simeq \hat{G}(\beta, k; \boldsymbol{\xi}'^{(1)}; \boldsymbol{\xi}^{(1)}) + \mathbf{s} \cdot \nabla' \hat{G}(\beta, k; \boldsymbol{\xi}'^{(1)}; \boldsymbol{\xi}^{(1)}),
\end{aligned} \tag{A.1}$$

where we have expanded around the lower grating's front pin position vector $\boldsymbol{\xi}^{(1)} = \mathbf{0}$. The notation $'$ is used to distinguish between the arguments in both the expansions and the directional derivatives; ∇ denotes that we are differentiating with respect to $\boldsymbol{\xi}^{(1)}$, whereas ∇' signifies differentiation with respect to $\boldsymbol{\xi}'^{(1)}$.

Let us consider the directional derivative terms in (A.1), which require differentiation of the Green's function (2.4). Define the function for the argument:

$$\rho_I(j, \boldsymbol{\xi}^{(1)}) = \left| ja\mathbf{e_1} + \boldsymbol{\xi}'^{(1)} - \boldsymbol{\xi}^{(1)} \right| = \sqrt{\left(ja + \xi_1'^{(1)} - \xi_1^{(1)} \right)^2 + \left(0 + \xi_2'^{(1)} - \xi_2^{(1)} \right)^2}. \tag{A.2}$$

Referring to (A.1), (A.2), for $\boldsymbol{\xi}^{(1)} = \boldsymbol{\xi}'^{(1)} = \mathbf{0}$, $\rho_I = |j|a$, we obtain

$$\left. \frac{\partial \hat{G}}{\partial \xi_1^{(1)}} \right|_{\boldsymbol{\xi}'^{(1)} = \boldsymbol{\xi}^{(1)} = \mathbf{0}} = \frac{-ja}{\rho_I} \left. \frac{\partial \hat{G}}{\partial \rho_I} \right|_{\boldsymbol{\xi}'^{(1)} = \boldsymbol{\xi}^{(1)} = \mathbf{0}} = - \left. \frac{\partial \hat{G}}{\partial \rho_I} \right|_{\boldsymbol{\xi}'^{(1)} = \boldsymbol{\xi}^{(1)} = \mathbf{0}}; \qquad \left. \frac{\partial \hat{G}}{\partial \xi_2^{(1)}} \right|_{\boldsymbol{\xi}'^{(1)} = \boldsymbol{\xi}^{(1)} = \mathbf{0}} = 0. \tag{A.3}$$

Using equations (A.1) and (A.3), we deduce

$$\begin{aligned}
\hat{G}_{12} &= \hat{G}(\beta, k; \boldsymbol{\xi}'^{(1)}; \boldsymbol{\xi}^{(2)}) \simeq \hat{G}(\beta, k; \boldsymbol{\xi}'^{(1)}; \boldsymbol{\xi}^{(1)}) + \mathbf{s} \cdot \nabla \hat{G}(\beta, k; \boldsymbol{\xi}'^{(1)}; \boldsymbol{\xi}^{(1)}) \qquad (A.4) \\
&\simeq \frac{i}{8\beta^2} \sum_{j=-\infty}^{\infty} \left[H_0^{(1)}(\beta|j|a) + \frac{2i}{\pi} K_0(\beta|j|a) + \beta s_1 \left(H_1^{(1)}(\beta|j|a) + \frac{2i}{\pi} K_1(\beta|j|a) \right) \right] e^{ikja}.
\end{aligned}$$

Similarly,

$$\begin{aligned}
\hat{G}_{21} &= \hat{G}(\beta, k; \boldsymbol{\xi}'^{(2)}; \boldsymbol{\xi}^{(1)}) \simeq \hat{G}(\beta, k; \boldsymbol{\xi}'^{(1)}; \boldsymbol{\xi}^{(1)}) + \mathbf{s} \cdot \nabla' \hat{G}(\beta, k; \boldsymbol{\xi}'^{(1)}; \boldsymbol{\xi}^{(1)}) \qquad (A.5) \\
&\simeq \frac{i}{8\beta^2} \sum_{j=-\infty}^{\infty} \left[H_0^{(1)}(\beta|j|a) + \frac{2i}{\pi} K_0(\beta|j|a) - \beta s_1 \left(H_1^{(1)}(\beta|j|a) + \frac{2i}{\pi} K_1(\beta|j|a) \right) \right] e^{ikja}.
\end{aligned}$$

We note that the expressions for $\hat{G}_{12}$ and $\hat{G}_{21}$ differ only by a change in sign for the sum of the first order Bessel functions, and that the terms are independent of s_2. This is unsurprising since we are expanding around $\boldsymbol{\xi}^{(1)}$ on the x-axis, but the approximation is only valid for sufficiently small $|\mathbf{s}|$. One may also consider the dipole approximation associated with expanding around the vector $\boldsymbol{\xi}^d$ halfway between the two gratings, which yields the same expressions as for the lower grating expansions (A.4), (A.5).

In Fig. A.1, we illustrate the efficacy of this approach for a typical example with $k_x = 1.1$ for two choices of the shift vector $\mathbf{s} = (0.05, 0.025)$, $\mathbf{s} = (0.01, 0.005)$. The exact values for the real parts of the G_{12} entry of the kernel matrix are plotted using a dashed (blue) curve, and those evaluated by the dipole-approximating function are plotted in solid (black), with $\mathbf{s} = (0.05, 0.025)$ in Fig. A.1(a), and $\mathbf{s} = (0.01, 0.005)$ in Fig. A.1(b). The improvement in the accuracy of the approximation for a smaller value of $|\mathbf{s}|$ is clearly evident in part (b).

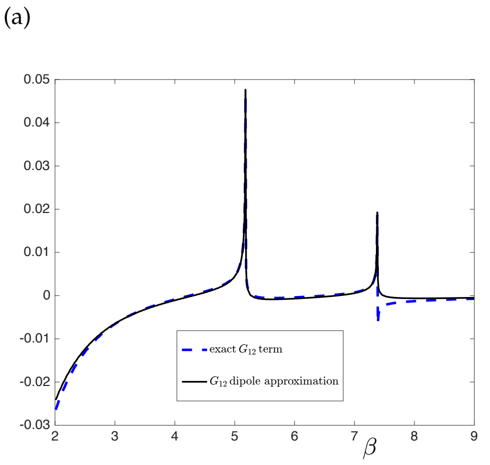

Figure A.1: Comparison of approximating function (solid black curve) for real parts of the G_{12} entries of the kernel matrix and their exact values (dashed blue curves) for $k_x = 1.1$ and β in the range $2 \leq \beta \leq 9$ for two shift vectors $\mathbf{s}$: (a) $\mathbf{s} = (0.05, 0.025)$, (b) $\mathbf{s} = (0.01, 0.005)$.

B. Evaluation of the coefficients for dipole approximation

Recalling equations (2.31), (2.32) we give the explicit expressions required to evaluate the coefficients S_n, D_n for a truncated semi-infinite grating (with truncation parameter L) using the wave scattering method. To determine $\mathbf{s} \cdot \nabla_{\boldsymbol{\xi}} g(\beta; \boldsymbol{\xi}; \boldsymbol{\xi}^{(1)})$, $\mathbf{s} \cdot \nabla_{\boldsymbol{\xi}^{(1)}} g(\beta; \boldsymbol{\xi}; \boldsymbol{\xi}^{(1)})$ we require the following functions and derivatives:

$$
\begin{aligned}
\rho_\xi &= \left[\left(\xi_1 - \xi_1^{(1)}\right)^2 + \left(\xi_2 - \xi_2^{(1)}\right)^2\right]^{\frac{1}{2}}, \quad \frac{\partial g}{\partial \rho_\xi} = -\frac{i}{8\beta}\left[H_1^{(1)}(\beta\rho_\xi) + \frac{2i}{\pi}K_1(\beta\rho_\xi)\right], \\
\frac{\partial \rho_\xi}{\partial \xi_k} &= \frac{\xi_k - \xi_k^{(1)}}{\rho_\xi}; \quad \frac{\partial \rho_\xi}{\partial \xi_k^{(1)}} = -\frac{(\xi_k - \xi_k^{(1)})}{\rho_\xi}. \qquad \text{(B.1)}
\end{aligned}
$$

For the additional term $\mathbf{s} \cdot \nabla_{\boldsymbol{\xi}} \left(\mathbf{s} \cdot \nabla_{\boldsymbol{\xi}^{(1)}} g(\beta; \boldsymbol{\xi}; \boldsymbol{\xi}^{(1)})\right)$ in (2.32), we also require the function $g_\xi^{(1)}$ and its derivative:

$$
g_\xi^{(1)} = H_1^{(1)}(\beta\rho_\xi) + \frac{2i}{\pi}K_1(\beta\rho_\xi); \; \frac{\partial g_\xi^{(1)}}{\partial \rho_\xi} = \beta H_0^{(1)}(\beta\rho_\xi) - \frac{H_1^{(1)}(\beta\rho_\xi)}{\rho_\xi} - \frac{2i}{\pi}\left[\beta K_0(\beta\rho_\xi) + \frac{K_1(\beta\rho_\xi)}{\rho_\xi}\right]. \qquad \text{(B.2)}
$$

Using the above formulae, equation (2.31) becomes

$$
\begin{aligned}
-e^{i\beta j a \cos\psi} &= \frac{i}{8\beta^2}\sum_{n=0}^{L-1}\left\{S_n\left[H_0^{(1)}(\beta|j-n|a) + \frac{2i}{\pi}K_0(\beta|j-n|a)\right]\right. \qquad \text{(B.3)} \\
&+ \left. D_n\frac{\beta s_1(j-n)a}{|j-n|a}\left[H_1^{(1)}(\beta|j-n|a) + \frac{2i}{\pi}K_1(\beta|j-n|a)\right]\right\}, \quad j = 0, 1, 2, \ldots
\end{aligned}
$$

Similarly, equation (2.32) becomes

$$
\begin{aligned}
- \quad & i\beta e^{i\beta j a\cos\psi}\left[s_1\cos\psi+s_2\sin\psi\right]=\frac{i}{8\beta^2}\Bigg(\sum_{n=0}^{L-1}-S_n\bigg\{\frac{\beta s_1(j-n)a}{|j-n|a}\bigg[H_1^{(1)}\left(\beta|j-n|a\right)\\
+ \quad & \frac{2i}{\pi}K_1\left(\beta|j-n|a\right)\bigg]\bigg\}+\sum_{n=0}^{L-1}D_n\bigg\{s_1^2\bigg(\beta^2\left[H_0^{(1)}\left(\beta|j-n|a\right)-\frac{2i}{\pi}K_0\left(\beta|j-n|a\right)\right]\\
- \quad & \frac{\beta}{|j-n|a}\left[H_1^{(1)}\left(\beta|j-n|a\right)+\frac{2i}{\pi}K_1\left(\beta|j-n|a\right)\right]\bigg)+\frac{\beta s_2^2}{|j-n|a}\bigg[H_1^{(1)}\left(\beta|j-n|a\right)\\
+ \quad & \frac{2i}{\pi}K_1\left(\beta|j-n|a\right)\bigg]\bigg\}\Bigg), \qquad j=0,1,2,\dots
\end{aligned}
\tag{B.4}
$$

We can express this system of equations in matrix form:

$$
\begin{pmatrix}\mathbf{F}^{(1)}\\ \mathbf{F}^{(2)}\end{pmatrix}=\frac{i}{8\beta^2}\begin{pmatrix}\mathbf{M}^{(1)} & \mathbf{M}^{(2)}\\ -\mathbf{M}^{(2)} & \mathbf{M}^{(3)}\end{pmatrix}\begin{pmatrix}\mathbf{S}\\ \mathbf{D}\end{pmatrix}, \tag{B.5}
$$

where $\mathbf{M}^{(1)}, \pm\mathbf{M}^{(2)}, \mathbf{M}^{(3)}$ are $L\times L$ block matrices and $\mathbf{F}^{(1)}, \mathbf{F}^{(2)}, \mathbf{S}, \mathbf{D}$ are $L\times 1$ column vectors with entries:

$$
\begin{aligned}
M_{jn}^{(1)} &= H_0^{(1)}\left(\beta|j-n|a\right)+\frac{2i}{\pi}K_0\left(\beta|j-n|a\right),\\
M_{jn}^{(2)} &= \frac{\beta s_1(j-n)a}{|j-n|a}\left[H_1^{(1)}\left(\beta|j-n|a\right)+\frac{2i}{\pi}K_1\left(\beta|j-n|a\right)\right],\\
M_{jn}^{(3)} &= s_1^2\bigg(\beta^2\left[H_0^{(1)}\left(\beta|j-n|a\right)-\frac{2i}{\pi}K_0\left(\beta|j-n|a\right)\right]-\frac{\beta}{|j-n|a}\bigg[H_1^{(1)}\left(\beta|j-n|a\right)\\
&\quad +\frac{2i}{\pi}K_1\left(\beta|j-n|a\right)\bigg]\bigg)+\frac{\beta s_2^2}{|j-n|a}\left[H_1^{(1)}\left(\beta|j-n|a\right)+\frac{2i}{\pi}K_1\left(\beta|j-n|a\right)\right];\\
F_j^{(1)} &= -e^{i\beta j a\cos\psi}, \qquad F_j^{(2)}=-i\beta e^{i\beta j a\cos\psi}\left[s_1\cos\psi+s_2\sin\psi\right].
\end{aligned}
\tag{B.6}
$$

To solve this system for a given incident field, the Bessel function derivatives require evaluation, in particular for the $\mathbf{M}^{(3)}$ terms which introduce logarithmic singularities for the cases $j=n$ (the main diagonal entries in the block matrices). The first derivative expressions in $\mathbf{M}^{(2)}$ have leading order terms:

$$
\frac{i\beta\rho_\xi[-i\pi+4\ln\left(\beta\rho_\xi\right)-4\ln 2-2+4\gamma]}{2\pi}+\mathcal{O}\left(\left(\beta\rho_\xi\right)^3\right), \quad \rho_\xi=|j-n|a,
$$

where γ represents the Euler constant, and which vanish for $j=n$. The second order derivatives may be expressed in the form:

$$
\frac{i\beta^2}{\pi}\left[2|\mathbf{s}|^2\left(\ln\left(\beta\rho_\xi\right)+\gamma-\ln 2+\frac{\pi}{4i}\right)+(s_1^2-s_2^2)\right]+\mathcal{O}\left(\left(\beta\rho_\xi\right)^2\right), \tag{B.7}
$$

which possesses a logarithmic term that grows slowly as $j\to n$. For sufficiently small $|\mathbf{s}|^2$, we approximate these terms using

$$
-\frac{1}{8\pi}\left[2|\mathbf{s}|^2\left(\ln\left(\frac{\beta|\mathbf{s}|}{2}\right)-4\beta|\mathbf{s}|+\gamma-\ln 2+\frac{\pi}{4i}\right)+(s_1^2-s_2^2)\right] \tag{B.8}
$$

for typical frequencies and shift vectors $\mathbf{s}$ that we consider here. The logarithmic term is replaced by the asymptotic term

$$
\ln\left(\frac{\beta|\mathbf{s}|}{2}\right)-4\beta|\mathbf{s}|.
$$

Note the change in the coefficient for (B.8) compared with (B.7), arising from the multiplication by the Green's function coefficient $i/(8\beta^2)$.

C. Derivation of algebraic system for herringbone

Recalling from equation (3.1) that the total flexural displacement $u(x, y)$ is given by

$$u(x,y) = u_{\text{inc}}(x,y) + \sum_{n=0}^{\infty} A_n^{(\text{I})} g(\beta; x, y; na_1, b/2) + \sum_{m=0}^{\infty} A_m^{(\text{II})} g(\beta; x, y; s_1 + ma_1, s_2 + b/2)$$
$$+ \sum_{c=0}^{\infty} A_c^{(\text{III})} g(\beta; x, y; ca_2, -b/2) + \sum_{d=0}^{\infty} A_d^{(\text{IV})} g(\beta; x, y; t_1 + da_2, t_2 - b/2), \quad \text{(C.1)}$$

the system of linear algebraic equations for the full pinned herringbone system is

$$- e^{i\beta[ja_1 \cos\psi + (b/2)\sin\psi]} = \sum_{n=0}^{\infty} A_n^{(\text{I})} g(\beta; ja_1, b/2; na_1, b/2)$$
$$+ \sum_{m=0}^{\infty} A_m^{(\text{II})} g(\beta; ja_1, b/2; s_1 + ma_1, s_2 + b/2) + \sum_{c=0}^{\infty} A_c^{(\text{III})} g(\beta; ja_1, b/2; ca_2, -b/2)$$
$$+ \sum_{d=0}^{\infty} A_d^{(\text{IV})} g(\beta; ja_1, b/2; t_1 + da_2, t_2 - b/2)$$
$$j = 0, 1, 2, \ldots \quad \text{(C.2)}$$

$$- e^{i\beta[(s_1+la_1)\cos\psi + (s_2+b/2)\sin\psi]} = \sum_{n=0}^{\infty} A_n^{(\text{I})} g(\beta; s_1 + la_1, s_2 + b/2; na_1, b/2) +$$
$$\sum_{m=0}^{\infty} A_m^{(\text{II})} g(\beta; s_1 + la_1, s_2 + b/2; s_1 + ma_1, s_2 + b/2) + \sum_{c=0}^{\infty} A_c^{(\text{III})} g(\beta; s_1 + la_1, s_2 + b/2; ca_2, -b/2)$$
$$+ \sum_{d=0}^{\infty} A_d^{(\text{IV})} g(\beta; s_1 + la_1, s_2 + b/2; t_1 + da_2, t_2 - b/2)$$
$$l = 0, 1, 2, \ldots \quad \text{(C.3)}$$

$$- e^{i\beta[pa_2 \cos\psi - (b/2)\sin\psi]} = \sum_{n=0}^{\infty} A_n^{(\text{I})} g(\beta; pa_2, -b/2; na_1, b/2)$$
$$+ \sum_{m=0}^{\infty} A_m^{(\text{II})} g(\beta; pa_2, -b/2; s_1 + ma_1, s_2 + b/2) + \sum_{c=0}^{\infty} A_c^{(\text{III})} g(\beta; pa_2, -b/2; ca_2, -b/2)$$
$$+ \sum_{d=0}^{\infty} A_d^{(\text{IV})} g(\beta; pa_2, -b/2; t_1 + da_2, t_2 - b/2)$$
$$p = 0, 1, 2, \ldots \quad \text{(C.4)}$$

$$-e^{i\beta[(t_1+qa_2)\cos\psi+(t_2-b/2)\sin\psi]}=\sum_{n=0}^{\infty}A_n^{(\mathrm{I})}g(\beta;t_1+qa_2,t_2-b/2;na_1,b/2)+$$

$$\sum_{m=0}^{\infty}A_m^{(\mathrm{II})}g(\beta;t_1+qa_2,t_2-b/2;s_1+ma_1,s_2+b/2)+\sum_{c=0}^{\infty}A_c^{(\mathrm{III})}g(\beta;t_1+qa_2,t_2-b/2;ca_2,-b/2)$$

$$+\sum_{d=0}^{\infty}A_d^{(\mathrm{IV})}g(\beta;t_1+qa_2,t_2-b/2;t_1+da_2,t_2-b/2)$$

$$q=0,1,2,\dots \quad \text{(C.5)}$$

The discrete Wiener-Hopf approach implemented in Section 2(b) is repeated here. We introduce similar notation for $N,M,C,D\in\mathbb{Z}$:

$$u(Na_1,b/2)=\begin{cases}0, & N\geq 0\\ B_N^{(\mathrm{I})}, & N<0\end{cases} \quad \text{(C.6)}$$

$$u(s_1+Ma_1,s_2+b/2)=\begin{cases}0, & M\geq 0\\ B_M^{(\mathrm{II})}, & M<0\end{cases} \quad \text{(C.7)}$$

$$u(Ca_2,-b/2)=\begin{cases}0, & C\geq 0\\ B_C^{(\mathrm{III})}, & C<0\end{cases} \quad \text{(C.8)}$$

$$u(t_1+Da_2,t_2-b/2)=\begin{cases}0, & D\geq 0\\ B_D^{(\mathrm{IV})}, & D<0\end{cases} \quad \text{(C.9)}$$

$$u_{\text{inc}}(Na_1,b/2)=F_N^{(\mathrm{I})}, \qquad u_{\text{inc}}(s_1+Ma_1,s_2+b/2)=F_M^{(\mathrm{II})}; \quad \text{(C.10)}$$

$$u_{\text{inc}}(Ca_2,-b/2)=F_C^{(\mathrm{I})}, \qquad u_{\text{inc}}(t_1+Da_2,t_2-b/2)=F_D^{(\mathrm{II})}. \quad \text{(C.11)}$$

Here $B_N^{(\mathrm{I})}$ to $B_D^{(\mathrm{IV})}$ represent the unknown amplitudes of the total flexural displacement at the points $(Na_1,b/2)$, $(s_1+Ma_1,s_2+b/2)$, $(Ca_2,-b/2)$ and $(t_1+Da_2,t_2-b/2)$ for, respectively, $N,M,C,D<0$ i.e. in the "reflection" region to the left of each grating pair. The field incident at the pins is denoted by $F_N^{(\mathrm{I})},F_M^{(\mathrm{II})},F_C^{(\mathrm{III})},F_D^{(\mathrm{IV})}$.

Similar to the preceding case of Section 2(b), we consider the displacement (C.1) at four field points $\mathbf{r}=(Na_1,b/2)$, $\mathbf{r}=(s_1+Ma_1,s_2+b/2)$, $\mathbf{r}=(Ca_2,-b/2)$ and $\mathbf{r}=(t_1+Da_2,t_2-b/2)$. As the definitions (C.6)-(C.11) suggest, the scattering coefficients are extended for $m,n,c,d<0$, where they are evaluated to be zero since there are no pins present in this region. Assuming the symmetric herringbone case ($a_1=a_2$), after applying the discrete Fourier Transform to equations (C.1)-(C.11) using Fourier variable k and indices of summation N, M, C and D, we obtain:

$$\sum_{N=-\infty}^{\infty} B_N^{(\mathrm{I})} e^{ikNa} = \sum_{N=-\infty}^{\infty} F_N^{(\mathrm{I})} e^{ikNa} + \sum_{n=-\infty}^{\infty} A_n^{(\mathrm{I})} e^{ikna} \sum_{j=-\infty}^{\infty} g(\beta; ja, b/2; 0, b/2) e^{ikja}$$
$$+ \sum_{m=-\infty}^{\infty} A_m^{(\mathrm{II})} e^{ikma} \sum_{j=-\infty}^{\infty} g(\beta; ja, b/2; s_1, s_2 + b/2) e^{ikja}$$
$$+ \sum_{c=-\infty}^{\infty} A_c^{(\mathrm{III})} e^{ikca} \sum_{j=-\infty}^{\infty} g(\beta; ja, b/2; 0, -b/2) e^{ikja}$$
$$+ \sum_{d=-\infty}^{\infty} A_d^{(\mathrm{IV})} e^{ikda} \sum_{j=-\infty}^{\infty} g(\beta; ja, b/2; t_1, t_2 - b/2) e^{ikja}, \quad \text{(C.12)}$$

$$\sum_{M=-\infty}^{\infty} B_M^{(\mathrm{II})} e^{ikMa} = \sum_{M=-\infty}^{\infty} F_M^{(\mathrm{II})} e^{ikMa} + \sum_{n=-\infty}^{\infty} A_n^{(\mathrm{I})} e^{ikna} \sum_{j=-\infty}^{\infty} g(\beta; s_1 + ja, s_2 + b/2; 0, b/2) e^{ikja}$$
$$+ \sum_{m=-\infty}^{\infty} A_m^{(\mathrm{II})} e^{ikma} \sum_{j=-\infty}^{\infty} g(\beta; s_1 + ja, s_2 + b/2; s1, s_2 + b/2) e^{ikja}$$
$$+ \sum_{c=-\infty}^{\infty} A_c^{(\mathrm{III})} e^{ikca} \sum_{j=-\infty}^{\infty} g(\beta; s_1 + ja, s_2 + b/2; 0, -b/2) e^{ikja}$$
$$+ \sum_{d=-\infty}^{\infty} A_d^{(\mathrm{IV})} e^{ikda} \sum_{j=-\infty}^{\infty} g(\beta; s_1 + ja, s_2 + b/2; t_1, t_2 - b/2) e^{ikja}, \quad \text{(C.13)}$$

$$\sum_{C=-\infty}^{\infty} B_C^{(\mathrm{III})} e^{ikCa} = \sum_{C=-\infty}^{\infty} F_C^{(\mathrm{III})} e^{ikCa} + \sum_{n=-\infty}^{\infty} A_n^{(\mathrm{I})} e^{ikna} \sum_{j=-\infty}^{\infty} g(\beta; ja, -b/2; 0, b/2) e^{ikja}$$
$$+ \sum_{m=-\infty}^{\infty} A_m^{(\mathrm{II})} e^{ikma} \sum_{j=-\infty}^{\infty} g(\beta; ja, -b/2; s_1, s_2 + b/2) e^{ikja}$$
$$+ \sum_{c=-\infty}^{\infty} A_c^{(\mathrm{III})} e^{ikca} \sum_{j=-\infty}^{\infty} g(\beta; ja, -b/2; 0, -b/2) e^{ikja}$$
$$+ \sum_{d=-\infty}^{\infty} A_d^{(\mathrm{IV})} e^{ikda} \sum_{j=-\infty}^{\infty} g(\beta; ja, -b/2; t_1, t_2 - b/2) e^{ikja}, \quad \text{(C.14)}$$

$$\sum_{D=-\infty}^{\infty} B_D^{(\mathrm{IV})} e^{ikDa} = \sum_{D=-\infty}^{\infty} F_D^{(\mathrm{IV})} e^{ikDa} + \sum_{n=-\infty}^{\infty} A_n^{(\mathrm{I})} e^{ikna} \sum_{j=-\infty}^{\infty} g(\beta; t_1 + ja, t_2 - b/2; 0, b/2) e^{ikja}$$
$$+ \sum_{m=-\infty}^{\infty} A_m^{(\mathrm{II})} e^{ikma} \sum_{j=-\infty}^{\infty} g(\beta; t_1 + ja, t_2 - b/2; s_1, s_2 + b/2) e^{ikja}$$
$$+ \sum_{c=-\infty}^{\infty} A_c^{(\mathrm{III})} e^{ikca} \sum_{j=-\infty}^{\infty} g(\beta; t_1 + ja, t_2 - b/2; 0, -b/2) e^{ikja}$$
$$+ \sum_{d=-\infty}^{\infty} A_d^{(IV)} e^{ikda} \sum_{j=-\infty}^{\infty} g(\beta; t_1 + ja, t_2 - b/2; t_1, t_2 - b/2) e^{ikja}. \quad \text{(C.15)}$$

The resulting system is an extended version of that for the shifted pair presented in Section 2(b).

D. Waveguide modes

In Table 1 we show computed values for the optimal grating separation b^* for a waveguide consisting of an unshifted pair of infinite gratings. The parameter η^* represents an idealised grating separation for first order waveguide modes, determined by the approximate waveguide model for the Helmholtz operator (neglecting the evanescent modes for the biharmonic case) and by (3.17). For gratings with unit periodicity ($a = 1$) and an incident plane wave characterised by ψ, the optimised spectral parameter value β^* is obtained for maximum single grating reflectance. Using equation (3.18), a corresponding k_x^* value is obtained. The various pairs of (β^*, k_x^*) values are used to determine resonant trapped modes, with excellent agreement with the approximate waveguide model, as shown by columns 4 and 5 of Table 1.

Table 1: Resonant frequencies β^* and wavenumbers k_x^*, and the corresponding optimised grating separation b^*, and idealised grating separation η^* for first order waveguide modes, for pairs of unshifted infinite gratings for various angles of incidence ψ.

ψ	β^*	k_x^*	η^*	b^*
0	4.456001	0	0.705025	0.705251
$\pi/60$	4.438147	0.232275	0.708833	0.709101
$\pi/30$	4.387466	0.458615	0.719982	0.720367
$\pi/20$	4.311191	0.674419	0.73779	0.738331
$\pi/15$	4.217801	0.87693	0.761482	0.762182
$\pi/12$	4.11476	1.06498	0.790427	0.79126
$\pi/10$	4.007707	1.23845	0.824228	0.825150
$7\pi/60$	3.900536	1.39783	0.862728	0.863689
$2\pi/15$	3.79580	1.54389	0.905975	0.906927
$3\pi/20$	3.6950925	1.67754	0.954209	0.955108
$\pi/6$	3.599363	1.79968	1.00784	1.00866
$11\pi/60$	3.509134	1.91121	1.06748	1.06818
$\pi/5$	3.424645	2.01296	1.133905	1.134490
$\pi/4$	3.205694	2.26677	1.38593	1.386185
$\pi/3$	2.94716	2.55232	2.131946	2.131958

E. Non-resonant example for dipole approximation of herringbone

Here we present a comparison of the scattering coefficients and flexural displacement fields for a non-resonant example of a herringbone system. For an incident plane wave characterised by $\psi = 0.805$ and $\beta = 3.92$, a symmetric herringbone system is defined by $\mathbf{t} = \mathbf{s}^- = (0.005, -0.015)$ with shift vector magnitude $|\mathbf{s}| = 0.016$ and spacing $b = 1.3$ (compare with Fig.11 in Section 3(d)(ii)). The real parts of the total displacement fields are plotted for a mid-section ($30 \le n \le 60$) for both the full herringbone system (four semi-infinite gratings) and the dipole approximation with two semi-infinite line arrays in, respectively, Figs. E.1(a), (b). The neighbourhoods of the front pins, illustrating the end effects, are shown in Figs. E.1(c), (d).

The changes in both phase and the scattering pattern are significantly reduced for this non-resonant example, when compared with the resonant case illustrated in Fig.11.

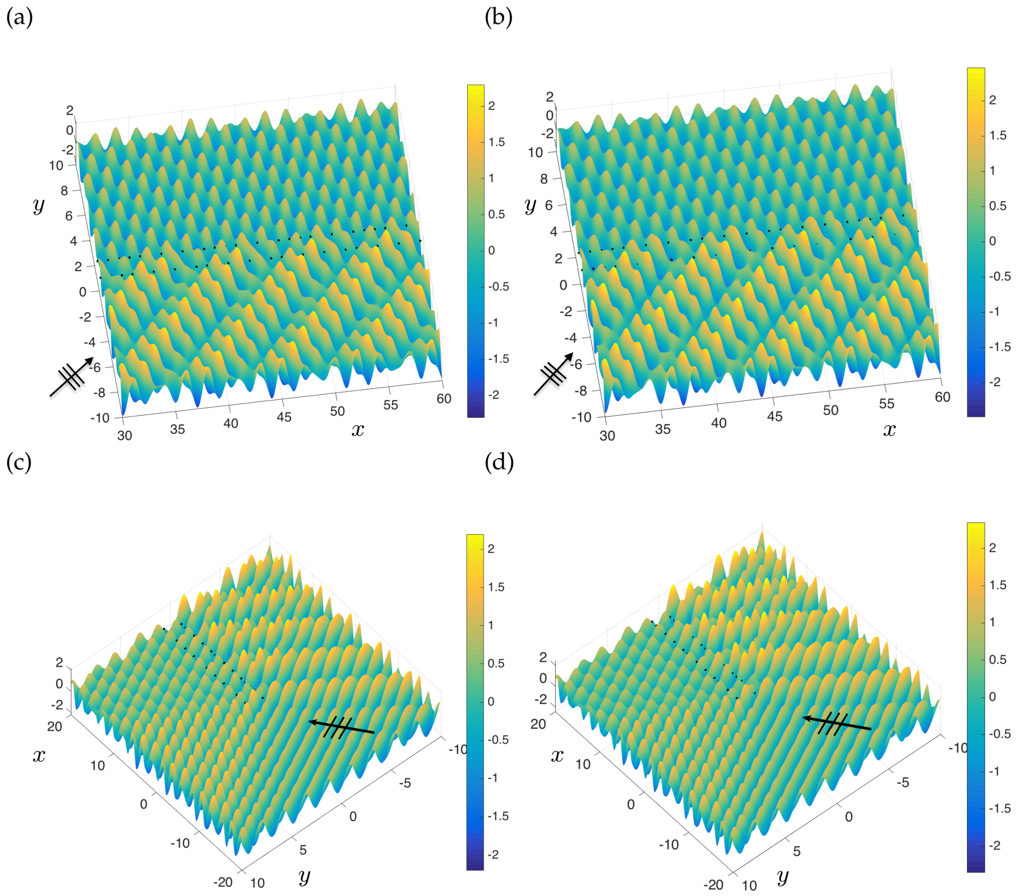

Figure E.1: Total displacement fields for the symmetric herringbone system with $|\mathbf{s}| = 0.016, b = 1.3$, $\mathbf{t} = \mathbf{s}^- = (0.005, -0.015)$ for $\psi = 0.805$ and $\beta = 3.92$, $L = 120$ for (a,c) herringbone, (b,d) dipole approximation.
